# NovumRNA: Accurate prediction of non-canonical tumor antigens from RNA sequencing data

**DOI:** 10.1016/j.isci.2025.113448

**Published:** 2025-09-03

**Authors:** Markus Ausserhofer, Dietmar Rieder, Manuel Facciolla, Raphael Gronauer, Giorgia Lamberti, Rebecca Lisandrelli, Serena Pellegatta, Zlatko Trajanoski, Francesca Finotello

**Affiliations:** 1 Department of Molecular Biology, Digital Science Center (DiSC), University of Innsbruck, 6020 Innsbruck, Tyrol, Austria; 2 Institute of Bioinformatics, Medical University of Innsbruck, Tyrol, Innsbruck, Austria; 3 Unit of Immunotherapy of Brain Tumors, Fondazione IRCCS Istituto Neurologico Carlo Besta, Milan, Italy

**Keywords:** cancer, genomics

## Abstract

Non-canonical tumor-specific antigens (ncTSAs) can expand the pool of targets for cancer immunotherapy, but require robust and comprehensive computational pipelines for their prediction. Here, we present NovumRNA, a fully automated Nextflow pipeline for predicting different classes of ncTSAs from patients’ RNA sequencing data. We extensively benchmarked NovumRNA using publicly available and newly generated datasets, demonstrating the robustness of its analytical modules and predictions. NovumRNA analysis of RNA-seq data from colorectal cancer organoids and patients’ tumor samples revealed comparable ncTSA potential for microsatellite stable and unstable tumors and candidate therapeutic targets for patients with low tumor mutational burden. Finally, our investigation of glioblastoma cell lines demonstrated increased ncTSAs burden upon indisulam treatment, and detection by NovumRNA of therapy-induced ncTSAs, which we could validate experimentally. These findings underscore the potential of NovumRNA for identifying synergistic drugs and novel therapeutic targets for immunotherapy, which could ultimately extend its benefit to a broader patient population.

## Highlights

• NovumRNA predicts non-canonical tumor antigens (ncTSAs) from patients’ RNA-seq data
• It is a robust standalone pipeline, extensively benchmarked on multiple datasets
• NovumRNA can identify new immunotherapy targets in hard-to-treat cancers
• Experimental validation confirms therapy-induced ncTSAs in glioblastoma

## Graphical abstract

**Figure:**
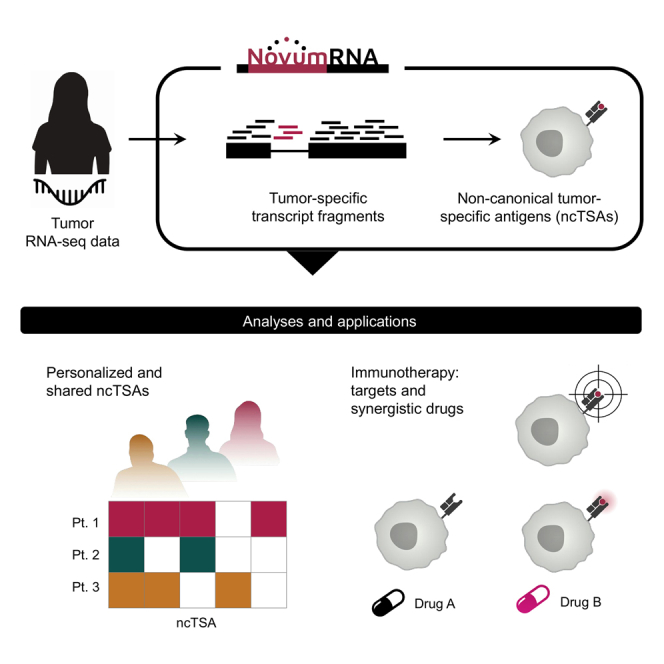

## Introduction

Immunotherapy has revolutionized cancer treatment, shifting the focus from targeting malignant cells to supporting the patient’s own immune system in eliminating cancer cells.^1^ Immunotherapy has achieved long-term durable responses in advanced cancers and is now approved worldwide for different tumor indications. Cancer immunotherapy broadly comprises immune checkpoint inhibitors (ICBs), personalized anticancer vaccines, and adoptive T cell therapies.^1^ Although different in their strategies, these modalities share the common goal of increasing the number and activity of T cells targeting neoantigens.^2^^,^^3^ Neoantigens are mutated peptides that are presented to the immune system by major histocompatibility complex (MHC) molecules (called human leukocyte antigen, HLA, in humans) on the surface of tumor cells.^3^^,^^4^^,^^5^ As they arise from non-synonymous single nucleotide variants (SNVs) and insertions or deletions (indels) present only in tumor cells and not in normal cells, neoantigens can elicit strong immune responses mediated by T cells, which recognize these peptides as “non-self.” Neoantigens are considered the major targets of immunotherapy responses, and their computational prediction in patients with cancer is the basis for personalized adoptive T cell therapy and anticancer vaccination.^3^^,^^6^^,^^7^ Therefore, the computational identification of personalized tumor neoantigens from patient’s next-generation sequencing (NGS) data is a key task in immuno-oncology.

Despite the success and potential of ICBs, the majority of patients still do not benefit from immunotherapy: either they are not eligible for it or do not respond.^8^^,^^9^ Therefore, there is a pressing need to identify a larger pool of tumor-specific targets to extend the population of patients with cancer treated with immunotherapy, and to understand and reverse the mechanisms of resistance to augment its clinical success. Tumor mutational burden (TMB), defined as the number of total non-synonymous mutations in a tumor, is a major predictive biomarker for immunotherapy response, as TMB-high tumors are more likely to generate neoantigens.^7^^,^^10^^,^^11^^,^^12^^,^^13^ Nevertheless, the correlation between TMB and immunotherapy outcome is far from perfect, and patients with low TMB have also shown responses to immunotherapy.^14^ For instance, renal cell cancer, despite having low TMB, has shown a good response rate to ICBs (∼25%).^14^ Neoadjuvant ICBs therapy has led to comparable pathological responses in patients with mismatch-repair-deficient and -proficient early-stage colorectal cancer (CRC).^14^^,^^15^

Altogether, these results suggest that immunotherapy can elicit immune responses targeting a broader pool of tumor-specific antigens (TSAs), including peptides generated from tumor aberrations that do not fall into the canonical definition of TMB. Indeed, recent studies have shown that TSAs can also arise from the expression of cancer-specific alternative-splicing events,^16^ gene fusions,^17^ expressed retroviral elements,^18^ and non-coding regions.^19^^,^^20^ These non-canonical TSAs (ncTSAs) can represent additional therapeutic targets whose potential is yet to be fully uncovered.^19^^,^^20^^,^^21^^,^^22^ Therefore, ncTSAs hold the promise to extend the benefit of immunotherapy to a wider population of patients with cancer, possibly targeting also low-TMB tumors, but their prediction from tumor NGS data requires ad hoc computational pipelines.

While there are several tools available for the prediction of neoantigens from SNV and indels, ncTSA prediction is still in its early stages. To date, there is no consensus on best practices and workflows for ncTSA prediction, and third-party benchmarking studies are lacking. However, computational pipelines and workflows have been recently proposed to predict certain classes of ncTSAs from patients’ NGS data.^19^^,^^23^^,^^24^^,^^25^^,^^26^^,^^27^^,^^28^^,^^29^^,^^30^^,^^31^^,^^32^ These methodologies founded a new toolkit for ncTSA-prediction, with each approach offering specific strengths (Table S1). However, none of these methods satisfies altogether a set of criteria that are required to warrant their broad adoption in research and clinical settings. First, providing comprehensive metadata about the predicted ncTSAs regarding their origin, genomic annotation, and, most importantly, likelihood to be presented to and recognized by T cells –information which is crucial for prioritization and experimental validation. Second, predicting ncTSAs from different types of non-canonical sources, as the best targets can depend on the specific tumor. Third, including predictions not only for class-I but also class-II ncTSAs, which are the targets of CD4^+^ T cell-mediated anticancer immunity.^33^^,^^34^ Fourth, implementing a dynamic and dedicated filtering approach to minimize false positives, namely *self-peptides* that are also expressed in non-cancerous tissues. Finally, offering an efficient, standalone, and well-documented software solution that can handle raw data inputs (without relying on third-party analytical tools), while ensuring ease of use and installation.

Here, we present NovumRNA: a Nextflow^35^ pipeline for the prediction of ncTSAs from raw tumor RNA sequencing (RNA-seq) data, addressing all the desired features outlined above. We confirmed NovumRNA predictions through the analysis of different RNA-seq datasets, demonstrating the robustness of its different analytical modules and final results. We further applied it to the analysis of a new RNA-seq dataset generated from ten tumor organoids derived from patients with microsatellite stable (MSS) and microsatellite unstable (MSI) CRC. With our analysis, we show how large control datasets can be used to more stringently ensure the tumor-specificity of the predicted ncTSA. Moreover, this analysis revealed a comparable ncTSA burden for both patients with MSS and MSI, and the identification of shared candidates across multiple patient-derived organoids, underlying the importance of further investigating the landscape of ncTSAs in hard-to-treat cancers. Analysis of two cancer cohorts from The Cancer Genome Atlas (TCGA)^36^ and the Therapeutically Applicable Research to Generate Effective Treatments (TARGET) initiatives further substantiated these findings, demonstrating that the prediction of ncTSAs can facilitate the identification of tumor-specific targets shared across patients with different cancer types. Finally, we generated an RNA-seq dataset from four patient-derived glioblastoma cell lines treated with the splicing-modulating drug indisulam. Using NovumRNA to analyze the data, we found a profound remodeling of the tumor-cell transcriptomes induced by this drug, which resulted in the generation of ncTSAs induced by the treatment. Using co-culture experiments, we experimentally validated NovumRNAs predictions, demonstrating its accuracy as well as its value for potentially pinpointing synergistic partners for immunotherapy.

## Results

### The NovumRNA pipeline allows the prediction of non-canonical tumor antigens from RNA sequencing data

**Figure 1:**
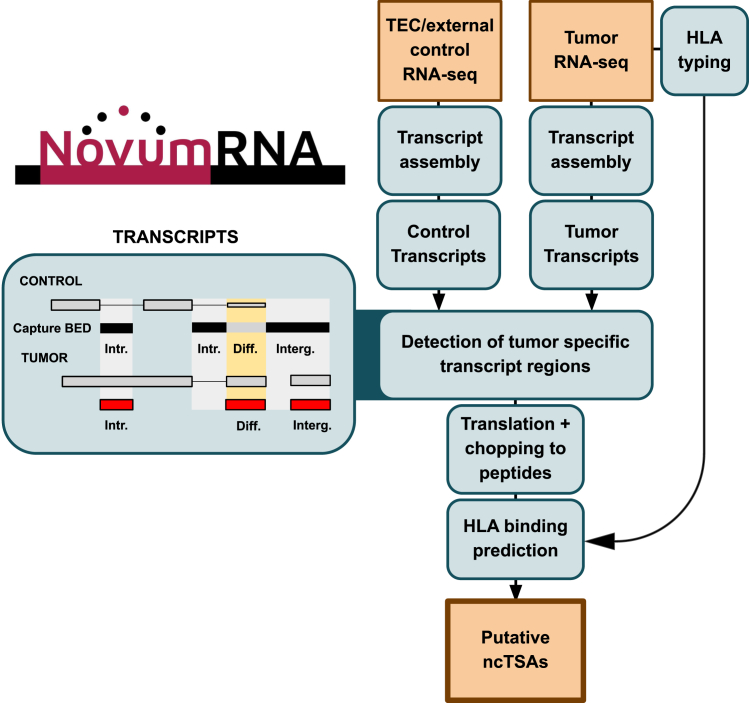
Schematization of the NovumRNA framework Tumor RNA sequencing FASTQ files (and, in parallel, if specified, non-cancerous control samples) are aligned to the reference genome to perform reference-guided transcript assembly. Tumor-specific and differentially expressed transcript fragments are identified by comparison with the control transcripts stored in the form of a capture BED file (derived from the internal thymic epithelial cell (TEC) reference or from external, user-provided control data). Filtered, tumor-specific transcript fragments are translated and fragmented into peptides of specified lengths. In parallel, patient-specific class-I and -II human leukocyte antigen (HLA) molecules are identified. Tumor-specific peptides are tested for their binding affinity toward the patient’s class-I and class-II HLA molecules. A final output data frame is generated containing all relevant metadata from each peptide, including endogenous retrovirus (ERV) annotation, to prioritize the best non-canonical tumor specific antigen (ncTSA) candidates.

NovumRNA is a multi-step, fully automated pipeline for the prediction of ncTSAs from human tumor RNA-seq data (Figure 1). It takes single- or paired-end RNA-seq reads (FASTQ files) as input, which are then aligned to the human reference genome using HISAT2,^37^ followed by reference-guided transcript assembly with StringTie.^38^

NovumRNA seeks to identify *tumor-specific* transcript fragments, defined as genomic regions that are exclusively covered by transcripts identified from the input tumor RNA-seq data and are instead completely absent in transcripts assembled from control data obtained from non-cancerous tissues. These control regions are identified by NovumRNA and stored in a capture BED file (details in STAR Methods). By default, regions annotated in the capture BED file are derived from an internal control database, which is based on 32 RNA-seq libraries from 8 samples of human thymic epithelial cells (TECs). As demonstrated in a previous study,^19^ TECs can be used as a normal control for the selection of TSAs thanks to their unique capability to express most known human genes and for their unique role in inducing T cell central tolerance to self-peptides. Alternatively, users can build their own filtering database, providing NovumRNA with a selection of control RNA-seq samples. Fragments that are strictly tumor-specific are considered *novel* and further annotated as *intronic* or *intergenic* based on their genomic location. Transcript fragments that have, instead, high expression levels in tumor cells and low expression levels in healthy controls are also retained for ncTSA prediction but annotated as *differential*.

Transcript fragments that pass the filtering steps (with user-customizable cut-offs) are then translated into peptide sequences. NovumRNA translates the full transcript of origin, either by using the corresponding reference protein extracted from GENCODE annotation,^39^ when available, or by selecting the reading frame based on the presence/location of methionine and/or Kozak motifs (details in STAR Methods). The translated fragments are then chopped into peptides of a specified length, which are additionally filtered against the GENCODE reference proteome to remove commonly known self-peptides.

In parallel, the patient’s class-I and -II human leukocyte antigen (HLA) molecules are identified from RNA-seq data using OptiType^40^ and HLA-HD,^41^ respectively. Alternatively, NovumRNA allows specifying the patient’s HLA types to be used for ncTSA prediction (skipping HLA typing). Finally, tumor-specific peptides are assessed for their binding affinity toward the patient’s class-I and -II HLA molecules using netMHCpan and netMHCIIpan,^42^ respectively.

NovumRNA reports ncTSAs of novel (intronic or intergenic) and differential origin. These ncTSAs are further categorized as endogenous retrovirus (ERV)-derived based on their overlap with known ERV regions according to the HERVd annotation.^43^ In addition to HLA-peptide binding affinity, NovumRNA provides a rich set of ncTSA-specific features relevant for prioritization and validation, including: exact genomic location of each predicted peptide, expression level of the original transcript, and different read-coverage statistics. In the prioritization of ncTSAs for clinical applications, it is key to select *high-quality* tumor-specific antigens that have a higher likelihood of immune recognition. As such, it is important that the underlying non-canonical tumor aberration: 1) is overrepresented in tumor cells rather than in healthy cells that can be captured by bulk-tumor RNA-seq, 2) it has high clonality,^2^^,^^44^ and 3) in the context of alternative splicing, it has a high percent spliced in (PSI). To this purpose, NovumRNA calculates a novel metric, called “TPM_iso_perc,” which is computed as the fraction of expression (in transcripts per millions, TPM) explained by the transcript from which a ncTSA derives from, relative to the sum of TPM expression across all other transcript isoforms.

NovumRNA is implemented in Nextflow DSL2^35^ and uses Singularity containers^45^ to allow for easy installation, parallelization, and customization. For additional information on NovumRNA methodology and usage, we refer to the STAR Methods section and to its GitHub repository (https://github.com/ComputationalBiomedicineGroup/NovumRNA).

### Impact of the control database on NovumRNA predictions

NovumRNA default filtering approach leverages a small but effective internal database of TEC-derived control transcripts to discriminate and filter out transcript sequences that are not tumor-specific (Figure 1). However, depending on the specific application, larger and/or tissue-specific control data might be preferred. NovumRNA offers a mode called “capture_bed,” where user-supplied RNA-seq data from healthy tissues can be used to create a new capture BED file, substituting the default TEC control database.

To demonstrate the value of this approach, we analyzed an RNA-seq dataset generated from ten CRC organoids (Table S2) using the default TEC filtering strategy, as well as an approach based on a capture BED file built from healthy-colon RNA-seq data obtained from the Genotype-Tissue Expression (GTEx) project.^46^ To assess the impact of the GTEx-based filtering approach, we performed a saturation analysis by randomly selecting an increasing number of GTEx colon samples for building the control database. In brief, the analysis was run with capture BED files made from 10 to 260 GTEx colon samples randomly selected from the whole pool of 262 (Table S3), repeating each sampling and corresponding analysis 10 times to also assess the robustness of the predictions (STAR Methods).

**Figure 2:**
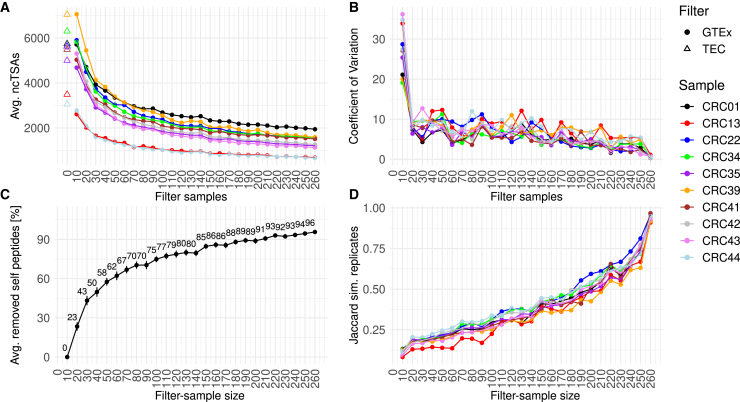
Effect of control database size on NovumRNA predictions on RNA sequencing data from colorectal cancer organoids In all panels, the x axis shows the number of healthy colon GTEx samples used for filtering. (A) Average number of predicted non-canonical tumor-specific antigens (ncTSAs). The triangles indicate the solution obtained using the default thymic epithelial cell (TEC) reference for filtering. (B) Coefficient of variation across replicates for the statistics shown in panel (A). (C) Percentage of filtered self peptides, considering the 10-sample filtering step as reference (i.e., 0% removal rate by definition). Dots represent the average across all organoids and replicates, while the vertical bars indicate the standard deviation. (D) Jaccard similarity of the predicted ncTSAs across all ten replicates for each organoid and filtering step.

As expected, the average number of predicted ncTSAs decreased as the number of healthy colon samples used for filtering (referred in the following to as “filter-sample size”) increased, with similar patterns across different organoids (Figure 2A). The default NovumRNA approach (based on 32 TEC RNA-seq libraries) predicted on average 70% more peptides compared to the GTEx solution at 30 samples (Figure 2A). The coefficient of variation (CV), indicating the variability of the predictions across the 10 replicates, was moderate when only ten GTEx samples were used (average 27%), but dropped rapidly already at 20 samples (average 8%) and decreased further as the filter-sample size grew (Figure 2B).

To evaluate the benefit of increasing the filter-sample size, we calculated the percentage of removed self-peptides at every filtering increment. Briefly, for every prediction, we identified putative self-peptides as the predicted ncTSAs that were not confirmed by the results obtained using the full GTEx dataset for filtering. Considering the 10-sample filtering step as a reference, we calculated the percentage of removed self-peptides at every filter-sample size, averaging across replicates and organoids (details in STAR Methods). Overall, filtering based on just 30 GTEx samples (i.e., ∼12% of the total GTEx data) already resulted in the removal of 43% of self peptides, while at 150 samples (∼58%), a removal rate of 85% was achieved (Figure 2C). To further assess filtering dynamics, we counted, for each filtering step, the number of ncTSAs unanimously predicted across all replicates, and quantified the robustness of the solution using the Jaccard similarity score. Starting with an average value of 0.11 across all organoids, similarity increased until 0.93 at the maximum filtering-sample size (Figure 2D). Overall, by using all 262 GTEx controls for filtering, the number of predicted ncTSAs decreased from 3,055–7,045 (median 5,568.5) identified with the default TEC filtering, to 657–1,759 (median 1,359).

### Comprehensive analysis of public datasets demonstrates the robustness of NovumRNA predictions

**Figure 3:**
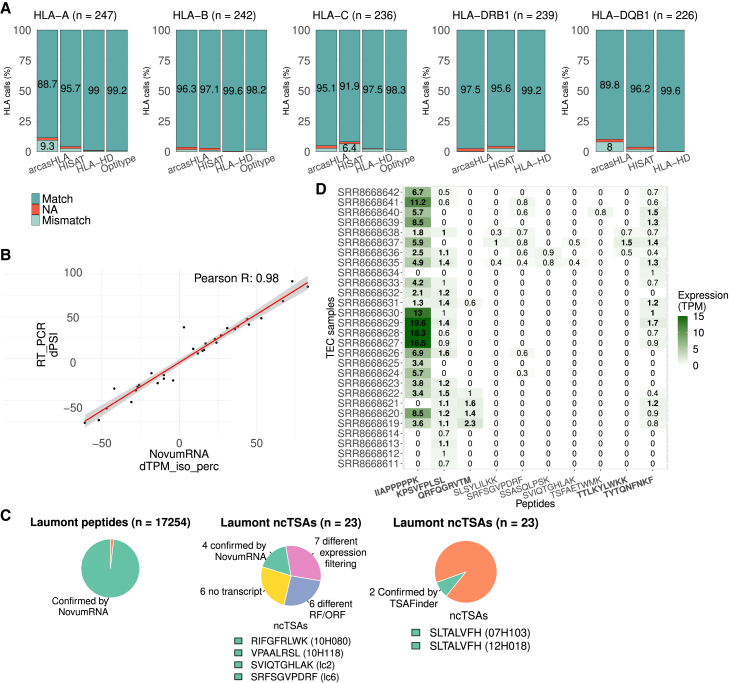
NovumRNA validation (A) Percentage of correct (“Match”), incorrect (“Mismatch”), and missing (“NA”) human leukocyte antigen (HLA) calls inferred by HLA-HD, HISATgenotype, arcasHLA (class I and II), and Optitype (for class-I genes), evaluated on RNA-seq data from the 1000 Genomes project. (B) Validation of NovumRNA “TPM_iso_perc” estimates using RNA-seq data from six cancer cell lines from Shen et al.^52^ Pearson correlation of delta exon inclusion rates from 32 out of 34 validated cassette-exons estimated by NovumRNA (“TPM_iso_perc,” x axis) vs. reverse transcription polymerase chain reaction (RT-PCR)-determined values (y axis). Gray, shaded area indicates the 95% confidence interval of the regression. (C) Validation of peptide and non-canonical tumor specific antigen (ncTSA) predicted with NovumRNA from Laumont et al.^19^ RNA-seq data. Left-to-right: pie charts show the fraction of peptides confirmed by NovumRNA (in green, 96.6%); number of ncTSAs confirmed (in green, with reported peptide sequence and sample identifier) and not confirmed by NovumRNA, or by TSAfinder. (D) Heatmap showing the expression in transcripts per million (TPM) in the control thymic epithelial cell (TEC) samples of peptides identified as tumor specific antigen (TSA) in the Laumont study. A “/” in the peptide sequence represents alternative amino acids (A/B), since the underlying nucleotide sequence showed a single nucleotide polymorphism (SNP) or mutation at this position.

To ensure the accuracy of the RNA-seq-based HLA typing module implemented in NovumRNA, we performed a benchmarking analysis of four state-of-the-art tools: arcasHLA,^47^ HISAT,^48^ HLA-HD^41^ (for class I and II), and OptiType (only class I).^40^ Building upon our previous work,^24^ we constructed a gold standard by combining data from two studies from the 1000 Genomes Project.^49^^,^^50^^,^^51^ In our assessment, all tools displayed high accuracy for both class-I and -II genes, confirming that HLA typing solely based on RNA-seq data is reliable (Figure 3A). HLA-HD and OptiType obtained the best performance on all class-I HLA genes, with percentage of correct calls ranging from 97.5% to 99.6% for HLA-HD, and 98.2% to 99.2% for OptiType. Notably, Optitype never resulted in a missing call (while HLA-HD has 0.4% missing calls); this feature has paramount importance for personalized cancer immunotherapy, where putative ncTSAs need to be predicted for every patient. For this reason and for its open-source licensing scheme, we based the class-I HLA typing module of NovumRNA on OptiType. On class-II HLA genes, HLA-HD performed best, with correct calls ranging between 99.2% and 99.6% (0.4% missing calls); therefore, class-II HLA typing in NovumRNA is performed with HLA-HD.

To assess NovumRNAs' effectiveness in detecting and quantifying differentially-spliced events from RNA-seq data –a daunting task even for specialized splicing-detection tools^53^– we considered a publicly available RNA-seq dataset from pancreatic cell lines, for which 34 cassette-exon events were experimentally confirmed via reverse transcription polymerase chain reaction (RT-PCR).^52^ NovumRNA confirmed 32 out of the 34 events, and its “TPM_iso_perc” estimates showed a high correlation with the differential percent spliced inclusion (dPSI) values based on RT-PCR measurements quantified in the original study (Pearson correlation of 0.98, *p* = 3 × 10^−21^) (Figure 3B).

To assess NovumRNAs' performance in ncTSA prediction, we used data from a study where putative ncTSAs were predicted from seven primary human tumor samples (Table 1) using a proteogenomic approach based on matched RNA-seq and mass spectrometry (MS) data.^19^ In this study, Laumont and colleagues made available for each sample, besides the transcriptomic data, the whole list of MS eluted peptides (*n* = 17,254), including ncTSAs (*n* = 23) identified via integrative analysis of RNA-seq and MS data. We applied NovumRNA to the tumor RNA-seq data, considering peptide lengths of 8–11 amino acids and using the default settings and the TEC control reference for the sake of comparability with the original study.

**Table 1:** Eluted peptides from the Laumont study confirmed by NovumRNA

Sample	HLA match	Peptides Laumont	Confirmed by NovumRNA	NovumRNA total ncTSAs	NovumRNAnovel ncTSAs
07H103 (B-ALL)	5/5	803	776 (96.6%)	14,828	10,635
10H080 (B-ALL)	4/4	3,110	3,045 (97.9%)	25,432	19,312
10H118 (B-ALL)	3/4	170	155 (91.1%)	22,212	16,743
12H018 (B-ALL)	6/6	371	357 (96.2%)	21,519	17,236
lc2 (Lung cancer)	5/5	5,288	5,202 (98.4%)	11,556	6,632
lc4 (Lung cancer)	4/4	4,616	4,551 (98.6%)	9,376	5,289
lc6 (Lung cancer)	4/4	2,889	2,838 (98.2%)	13,041	7,816

Shown are the total number of mass spectrometry (MS)-confirmed, eluted peptides from seven primary human cancer samples, as well as the number and percentage of peptides confirmed by NovumRNA, together with the proportion of matching human leukocyte antigen (HLA) type calls. B-ALL: B-lineage acute lymphoblastic leukemia.

We first compared class-I HLA types predicted with OptiType by NovumRNA with those reported in the Laumont study (Table 1). All HLA types agreed at four-digit resolution, except for one HLA allele for sample 10H118, which agreed on only at two-digit resolution (HLA-C∗07:18/07:37 NovumRNA vs. HLA-C∗07:01 Laumont) (Table 1).

We further compared NovumRNA-predicted peptides to the list of MS-confirmed, eluted peptides (*n* = 17,247). Since NovumRNA, by default, only translates transcripts containing relevant tumor-specific fragments, the translation module was run separately from the pipeline on all transcripts assembled by StringTie, and the resulting proteins were fragmented into all possible 8–11 amino acid-long peptides. On average, 96.6% of eluted peptides were confirmed by NovumRNA for each sample (Table 1; Figure 3C, left).

Finally, we compared the putative ncTSAs identified by NovumRNA (predicted solely from RNA-seq data) with the ones obtained in the original study from the joint analysis of RNA-seq and MS data. From the total 23 ncTSAs identified in the Laumont study, 4 were confirmed also by NovumRNA (2 novel and 2 differential), including the confirmation of the correct HLA type (Table 2; Figure 3C middle). For comparison, a recent study focused on the prediction of ncTSAs from this dataset using a tool called TSAFinder^25^ could identify only a single matching peptide, present in two samples (SLTALVFHV, samples 07H103 and 12H018) (Figure 3C, right).

**Table 2:** Comparison of NovumRNA and Laumont ncTSA predictions

Sample	ncTSA	ncTSA region reconstructed by StringTie	maxTPM in control ≤ 1	NovumRNA transcript class	minTPM in tumor >10 (Differential only)	minTPM in tumor ≥ 1 (Novel only)	Correct RF	Correct ORF	HLA type match
07H103	KILILLQSL	**YES**	–	Novel	–	**6.9**	NO	–	–
07H103	KISLYLPAL	NO	–	–	–	–	–	–	–
07H103	SLTALVFHV	**YES**	–	Novel	–	**876.4**	NO	–	–
10H080	HETLRLLL	NO	–	–	–	–	–	–	–
10H080	TSIPKPNLK	**YES**	–	Novel	–	**1.7**	**YES**	NO	–
10H080	**RIFGFRLWK**	**YES**	–	**Novel**	–	**7.4**	**YES**	**YES**	**YES**
10H080	TSFAETWMK	NO	**0.8**	–	–	–	–	–	–
10H118	LPFEQKSL	NO	–	–	–	–	–	–	–
10H118	SLREKGFSI	NO	–	–	–	–	–	–	–
10H118	**VPAALRSL**	**YES**	–	**Novel**	–	**1.5**	**YES**	**YES**	**YES**
12H018	LLAATILLSV	NO	–	–	–	–	–	–	–
12H018	SLTALVFHV	**YES**	–	Novel	–	1925.3	NO	–	–
lc2	IIAPPPPPK	**YES**	19.6	–	–	–	–	–	–
lc2	LVFNIILHR	**YES**	–	Novel	–	0.4	–	–	–
lc2	MISPVLALK	**YES**	–	Novel	–	**43.5**	**YES**	NO	–
lc2	SLSYLILKK	**YES**	1	Differential	0.3	–	–	–	–
lc2	SSASQLPSK	**YES**	**0.9**	Differential	133.3	–	NO	–	–
lc2	**SVIQTGHLAK**	**YES**	**0.5**	**Differential**	**48.4**	–	**YES**	**YES**	**YES**
lc2	TTLKYLWKK	**YES**	1.5	–	–	–	–	–	–
lc4	KPSVFPLSL	**YES**	1.6	–	–	–	–	–	–
lc6	QR/KF/LQGRVTM	**YES**	2.3	–	–	–	–	–	–
lc6	**SRFSGVPDRF**	**YES**	**0.8**	**Differential**	**1475.8**	–	**YES**	**YES**	**YES**
lc6	TYTQN/DFNKF	**YES**	1.7	–	–	–	–	–	–

Table shows the features of non canonical tumor specific antigens (ncTSAs) identified in the Laumont study that were confirmed (in bold) or not confirmed by NovumRNA according to the adopted filtering approach. The “ncTSA region” column indicates whether the ncTSA region was reconstructed by StringTie. The “maxTPM” column indicates whether the maximum expression in the control samples did not exceed 1 transcrip per million (TPM). The “NovumRNA class” reports NovumRNA-determined ncTSA classes, as “novel” or “differential.” For “differential” ncTSAs, the minimum level of expression in (“minTPM”) in the tumor sample must be greater than 10 TPM. For “novel” ncTSAs, “minTPM” should be greater than or equal to 1 TPM. The “Correct RF” and “Correct ORF” columns indicate if the correct reading frame (RF) and open reading frame (ORF) were predicted by NovumRNA. Finally, the “HLA type match” column indicates if the peptide-bound human leukocyte antigen (HLA) type predicted by NovumRNA matches that identified in the study. A “/” in the peptide sequence represents an alternative amino acid, since the underlying nucleotide sequence showed a single nucleotide polymorphism (SNP) or a point mutation at this position.

Given the differences in terms of used input data and analytical approach, we investigated at which point of the analysis NovumRNA and Laumont ncTSA predictions diverged. As the location (chromosome plus start and stop position) of all the 23 ncTSAs were available, we checked if these genomic regions were covered by the transcripts assembled by the StringTie module of NovumRNA (Table 2). Of the 23 ncTSA regions, 17 were successfully reconstructed, while six were undetected (Table 2; Figure 3C, middle).

For an ncTSA to be classified as “tumor-specific” by NovumRNA, its maximum expression across control samples must not exceed a certain threshold, set at 1 TPM by default. Despite being labeled as “tumor-specific” in the original study, we found that seven ncTSAs were expressed in the TEC controls (Figure 3D). Three of them showed non-negligible expression in multiple samples, exceeding the 1 TPM cut-off: IIAPPPPPK (max: 19.6 TPM), KPSVFPLSL (max: 1.6 TPM), and TTLKYLWKK (max: 1.5 TPM) (Table 2; Figure 3D in bold). Given these expression levels in the controls, NovumRNA did not select these three peptides as ncTSAs (Table 2). Two more ncTSAs, QR/KF/LQGRVTM (max: 2.3 TPM) and TYTQN/DFNKF (max: 1.7 TPM), this time with a reported expression in the controls also in the original study, also exceeded NovumRNA cut-offs and were filtered out. From the remaining peptides, one differential (SLSYLILKK) and one novel (LVFNIILHR) putative ncTSA were instead discarded by NovumRNA due to their low expression in the tumor samples (Table 2).

Finally, six of the remaining putative ncTSAs were not identified by NovumRNA due to a different prediction of their reading frame (RF) or open reading frame (ORF). Of note, NovumRNA was able to predict the correct RF for 6 over 10 peptides, and the correct ORF for 4 over 10 peptides (Table 2) without relying on 3-frame translation or additional proteomics data as done in the original study. Due to the complexity of RF and ORF identification from de novo-assembled transcripts, we tested an alternative approach based on BORF, a tool specifically designed for ORF prediction.^54^ The usage of BORF in the NovumRNA framework led to the identification of one additional ncTSA (SLTALVFH, present in two samples), counterbalanced by the loss of one ncTSA found by NovumRNA (RIFGFRLWK).

As the total set of truly immunogenic peptides is not known for the Laumont, we could not use this dataset to assess NovumRNA precision. Nevertheless, to shed some light on the capability of NovumRNA to effectively filter out false positives (FPs), we considered as “negative hits” a set of MS-derived peptides that were not ultimately confirmed as TSAs in the original study. Leveraging this set of 17,224 *negative* peptides, we computed, for each sample, the number of peptides wrongly selected by NovumRNA as ncTSAs (Table S4). Of the total 167-to-5,281 negative peptides per sample, only 0-to-6 were selected by NovumRNA as ncTSAs, and none of those were among the novel ones.

Taken together, the results of our benchmarking efforts based on several publicly available datasets demonstrate the robustness of NovumRNA analytical modules and overall approach, especially underscoring its capability to effectively select novel ncTSAs.

### Prediction of non-canonical tumor antigens in colorectal cancer organoids and tumor samples identifies potential targets in microsatellite-stable tumors

**Figure 4:**
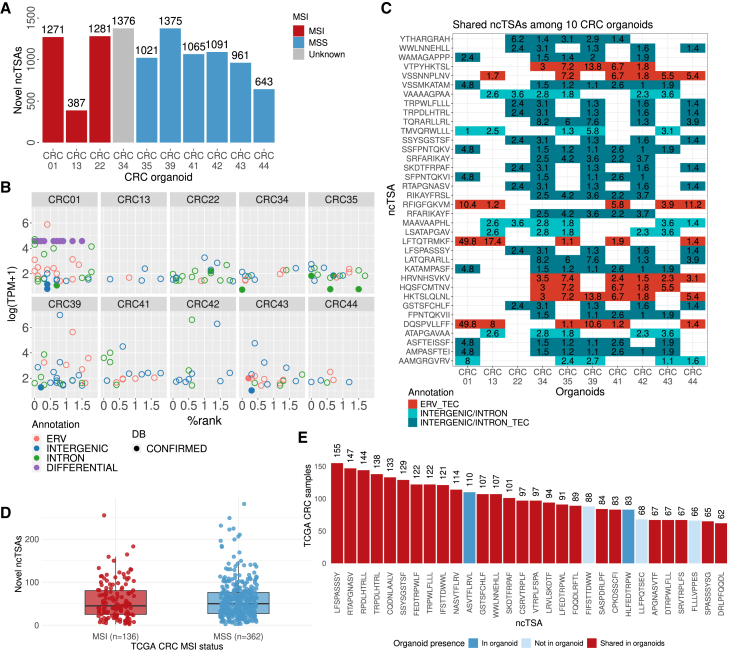
NovumRNA analysis of colorectal cancer organoids (A) Number of novel non-canonical tumor-specific antigens (ncTSAs) in microsatellite unstable (MSI) and microsatellite stable (MSS) colorectal cancer (CRC) organoids. (B) Top ncTSA candidates predicted by NovumRNA for all ten organoids, with reported binding affinity as % rank (x axis), expression level as log-scaled transcripts per million (TPM) (y axis), ncTSA class (indicated by the dot color). Database hits are indicated as full dots. (C) Heatmap showing the top-shared ncTSAs across CRC organoids (shared by at least five organoids and originating from multi-exon transcripts). Tiles are colored by ncTSA origin annotation; darker colors indicate whether the ncTSA were also found using the default thymic epithelial cell (TEC) filtering approach. The numbers in tiles indicate the TPM expression of the transcript that the ncTSAs derive from. (D) Boxplot showing the number of novel ncTSAs in MSI and MSS samples from The Cancer Genome Atlas (TCGA) CRC cohort (*n* = 499). (E) Top 30 shared ncTSAs from multi-exon transcripts in TCGA CRC samples (*n* = 499). The bars quantify in how many samples each peptide was found and are colored according to their presence in CRC-organoid results: red (top-shared ncTSA in CRC organoids), blue (in organoids, not shared), and light blue (not in organoids).

Leveraging NovumRNA predictions obtained from CRC organoid data (using all 262 GTEx controls for filtering), we investigated the ncTSA landscape in three MSI and six MSS tumors. Overall, there were no significant differences between the MSI and MSS tumors in the total number of novel ncTSAs (*p* = 0.904, Wilcoxon rank-sum test, Figure 4A). Similarly, there were no significant differences for the individual ncTSA classes (Figure S1). The top ncTSA candidates, selected based on HLA binding affinity, transcript expression, coverage, and clonality/PSI (details in STAR Methods), represented all novel classes (Figure 4B; Table S5). By querying the identified peptides in public antigen databases, we found matches for 11 novel and 19 differential ncTSAs in four databases^55^^,^^56^^,^^57^ (Figure 4B; Table S6). We could further assign the sequences of six ERV-derived ncTSAs (TQSLFGGLF, SLFGGLFTR, LCSMRKIHL, ENYSQRGLF, CSMRKIHLR, FRLFLIRPH), three of which were shared across two organoids, to the ERVH-5 retroviral gene.

We further investigated if the predicted ncTSAs were shared across CRC organoids, focusing on peptides arising from multi-exon transcripts to limit the impact of possible FPs. 693 ncTSAs were shared across at least two organoids, while four were shared across seven organoids; no ncTSAs were shared across all ten organoids (Figure 4C; Table S7). Notably, 143/693 shared ncTSAs (20.6%) were derived from ERV sequences. Among all ncTSAs predicted using the whole GTEx dataset for filtering, 8,161 (65%) were also present among the final ncTSAs predicted using the default TEC filtering approach.

To further corroborate these results, we extended our ncTSA analysis to the CRC TCGA cohort (Table S8), identifying a total of 14,099 novel ncTSAs across 499 samples. Comparative analysis confirmed no significant differences in the average number of predicted ncTSAs between MSI and MSS tumors (mean = 56.5 vs. 58.6, *p* = 0.578, Wilcoxon rank-sum test) (Figure 4D). The average number of novel ncTSA was 45 for both MSS and MSI tumors. For reference, the average number of predicted SNV-derived canonical neoantigens was 336 and 630 for MSS and MSI, respectively, according to a previous study.^58^ We further investigated shared, multi-exon ncTSAs (Table S9): 2,685 ncTSAs (24.1%) were shared by at least two samples, and the top-30 shared ncTSAs were present in a range of 62-to-155 samples; of these, 25 were also identified as shared ncTSAs in the CRC organoids (Figure 4E).

### NovumRNA identifies non-canonical tumor neoantigens induced by indisulam treatment in glioblastoma cell lines

RNA-seq provides a cost-effective technique to investigate the dynamics of gene expression upon perturbation. NovumRNA can be effectively applied to perturbed RNA-seq data generated from treated cancer cell lines to investigate how drugs modulate the expression of ncTSAs, potentially influencing the immunogenicity of tumor cells. To test NovumRNA on this application, we analyzed an RNA-seq dataset generated from the human bladder cancer cell line UM-UC-3 treated with the methylation inhibitor 5-Aza-2′-deoxycytidine (5-Aza-CdR) and subjected to RNA-seq after 5, 9, 13, and 17 days of treatment.^59^ NovumRNA was applied with default parameter settings, considering binding peptides of 9 amino acids in length (STAR Methods). The highest number of novel ncTSAs was predicted at day 17 (Figure S2A), in accordance with the largest drug-induced transcriptional effect seen in the original study.^59^ To rule out the possibility that this pattern was due to sequencing depth bias, we computed Pearson correlation between the number of mapped reads and the total number of ncTSAs predicted per treatment, confirming no association (R = 0.41, *p* = 0.24, Figure S3).

We analyzed a second dataset from cell lines treated with the splicing-modulating drugs indisulam and MS023.^60^ In this study, RNA-seq data were generated from three human melanoma cell lines (510 MEL, A375, SK MEL 239), with triplicates for each cancer cell line, treated for 96 h with indisulam, MS023, or with dimethyl sulfoxide (DMSO), used as a control.

NovumRNA analysis revealed an increase in the number of predicted novel ncTSAs (i.e., intronic, intergenic, and ERV) in all cell lines and replicates treated with indisulam compared to the control group (Figure S2B). The number of ncTSAs of differential origin showed instead no change (510 MEL) or a decrease (SK MEL 239, A375) upon treatment. Compared to the A375 cell line, which showed moderate changes upon indisulam treatment, the SK MEL 239 and, especially, 510 MEL cell lines exhibited a marked augmentation in the number of predicted ncTSA in the treated vs. control condition: SK MEL 239: intronic (+150%, *p* = 0.03), intergenic (+140%, *p* = 0.05), ERV (+230%, *p* = 0.05, one-sided Wilcoxon rank-sum test); 510 MEL: intronic (+380%, *p* = 0.05), intergenic (+230%, *p* = 0.05), ERV (+330%, *p* = 0.05). Treatment with MS023 resulted in no significant changes in terms of ncTSA numbers, with the exception of intronic ncTSAs in the 510 MEL cell line (+118%, *p* = 0.05) (Figure S2B).

The profound changes in ncTSA potential fostered by indisulam treatment motivated us to investigate the effect of this drug in a non-immunogenic cancer: glioblastoma. Primary cell lines were generated from surgical material collected from four distinct patients with glioblastoma and grown *in vitro* as neurospheres (GB-NS, Table S10). GB-NS were then treated with indisulam or vehicle control and subjected to bulk RNA-seq (STAR Methods). The resulting data was analyzed with NovumRNA, using 120 healthy brain GTEx samples as a normal control for filtering (Table S3).

**Figure 5:**
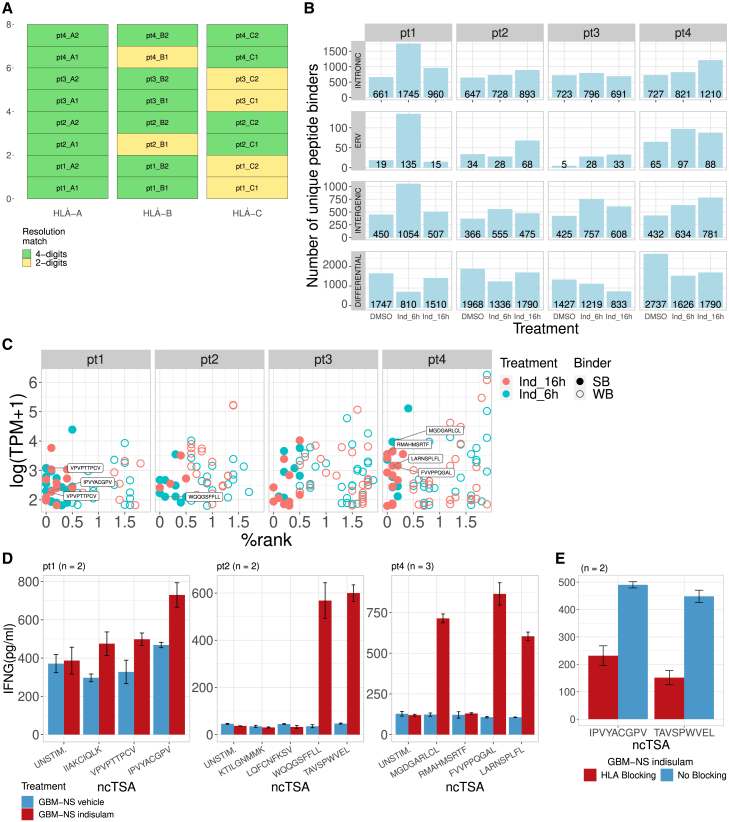
NovumRNA analysis and validation of glioblastoma-derived cell lines treated with indisulam (A) Experimental validation of Optitype-based class-I human leukocyte antigen (HLA) typing results. Green fields indicate a match on 4-digits resolution for a specific patient’s sample and HLA allele, while yellow fields indicate an agreement at 2-digit resolution (due to the lower resolution of the polymerase chain reaction (PCR)-based HLA typing). (B) Bar plots showing the number of different classes of non-canonical tumor specific antigens (ncTSAs) (unique peptide binders) predicted by NovumRNA for the four patients’ cell lines, treated with indisulam for 6 or 16 h, or with dimethyl sulfoxide (DMSO), used as control. ncTSAs are split by their genomic origin. (C) Top ncTSA candidates predicted by NovumRNA for the four patients with glioblastoma, with reported binding affinity as % rank (x axis), expression level as log-scaled transcripts per millions (TPM, y axis), and time point (indicated by the dot color). The labels indicate the peptide sequence of the ncTSAs selected for validation. (D) Bar plots summarizing the results of the T-cell essay testing the immunogenicity of the selected ncTSAs. Interferon gamma (IFN-γ) secretion levels in peripheral blood mononuclear cells (PBMCs) co-cultured with tumor cells treated with indisulam (red) or DMSO (blue). PBMCs were either unstimulated (“UNSTIM.”) or pre-stimulated with the selected ncTSAs predicted via NovumRNA (labeled in panel C). Error bars represent the standard deviation. (E) Bar plots show the results of the T cell assay performed for one selected immunogenic ncTSA identified in panel D for patients 1 and 2. IFN-γ secretion levels were measured in pre-stimulated PBMCs co-cultured with tumor cells treated with indisulam, with or without HLA-blocking antibody. Error bars represent the standard deviation.

In addition to our in silico analysis, we performed a series of experiments to confirm NovumRNA predictions. First, we validated NovumRNA class-I HLA typing results using polymerase chain reaction (PCR). We could experimentally determine the HLA alleles at 4-digit resolution for all patients and genes, except for 2 and 4 HLA-B and HLA-C alleles, respectively, which were identified at 2-digit resolution; all the PCR-determined HLA alleles matched the predictions made by NovumRNA (Figure 5A, HLA types not reported due to privacy considerations).

Then, we investigated the reshaping of the ncTSA glioblastoma landscape induced by indisulam. Indisulam treatment of GB-NS cells resulted in an increase of novel ncTSAs (ERV, intergenic, and intronic) predicted by NovumRNA across the four samples, with peaks that varied in latency depending on the patient’s sample (Figure 5B). Also, the magnitude of change upon indisulam treatment was patient-specific: the changes in the number of predicted ncTSA were particularly marked for patient 1 and limited instead for patient 3. Differential ncTSAs decreased across all patients upon indisulam treatment.

To validate NovumRNA predictions, we focused on a selection of *novel* candidates identified from the indisulam-treated samples and not the controls, and having a higher likelihood of being presented and recognized by T cells (Figure 5C; Table S11, details in STAR Methods). For this validation, we disregarded patient 3 due to homozygosity for all the three HLA genes (data not shown) and lower resolution available for HLA typing confirmation. We performed a co-culture experiment with autologous PBMCs, either unstimulated or pre-stimulated with the eleven peptides selected for validation, and GB-NS cells treated with indisulam or DMSO. This experiment resulted in an increased secretion of interferon gamma (IFN-γ), as evaluated by ELISA assay, after 24-h treatment with indisulam compared to the control for six out of the eleven evaluated peptides. Two peptides from patient 2 increased the average IFN-γ secretion upon treatment by 13- and 16-fold, respectively, while three peptides from patient 4 showed a 5-to-8-fold increase. The higher secretion of IFN-γ was consistent with an increased percentage of CD8^+^ T cells CD45RA/CD62L double-negative, indicating an effector memory-like phenotype, demonstrating their immunogenicity as predicted by NovumRNA (Figures 5D; Table S12). To confirm the HLA-restricted nature required for peptide recognition by T cells, IFN-γ secretion was re-evaluated with ELISA for two of the top immunogenic peptides in the presence of a specific HLA-blocking antibody. IFN-γ levels were significantly higher in the absence of HLA blockade (*p* = 0.014, one-sided Wilcoxon signed-rank test), supporting a T cell-mediated and HLA-dependent mechanism (Figure 5E).

## Discussion

In this study, we introduced NovumRNA, a computational pipeline for the prediction of human non-canonical tumor-specific antigens (ncTSAs) from tumor RNA-seq data. Recent findings have unveiled ncTSAs as targets of anticancer immune responses.^16^^,^^17^^,^^18^^,^^19^^,^^20^^,^^21^^,^^22^ Therefore, the ability to computationally predict these antigens from tumor NGS data could significantly broaden the reach of T-cell-mediated cancer immunotherapies, making them accessible and effective for a larger population of patients.

NovumRNA distinguishes itself from previous approaches for ncTSA prediction by being a standalone and fully automated software solution that can analyze raw tumor RNA-seq data without relying on third-party tools for data preprocessing. Thanks to its implementation based on Nextflow DSL2^35^ and Singularity containers,^45^ NovumRNA ensures portability, scalability, and reproducibility. NovumRNA predicts peptides derived from a wide range of non-canonical, tumor-specific alterations, such as alternative splicing events, expressed retroviral elements, and non-coding regions, and complements its predictions with extensive metadata for downstream neoepitope prioritization and validation. At present, NovumRNA does not predict peptides derived from gene fusions, a task for which we recommend using dedicated tools such as NeoFuse^23^ or pVACfuse.^28^ The current version of NovumRNA already takes into consideration single nucleotide polymorphisms (SNPs) supported by the majority of the aligned reads, which can alter the sequence of ncTSAs; future improvements will also support the handling of indels as well as of minor-allele SNPs. While our study focuses on class-I ncTSA prediction, we point out that NovumRNA also predicts class-II ncTSAs, which are crucial for the recognition of tumor cells by CD4^+^ T cells.^33^^,^^34^ Of note, as NovumRNA relies on transcriptomic evidence, it does not aim to detect ncTSAs originating from tumor-specific translational events, such as cryptic ORFs.^61^

In silico prediction of TSAs is challenging, even for neoantigens derived from sources such as SNV and indels,^6^^,^^44^^,^^62^ for which detailed best practices have been proposed.^63^^,^^64^ Nevertheless, we could demonstrate the robustness of NovumRNA modules and predictions using a diverse panel of publicly available and ad-hoc-generated datasets with associated gold standards. In particular, using RNA-seq data from a recent proteogenomic study,^19^ we could demonstrate a marked agreement of NovumRNA predictions with the original results and superior performance compared to the competitor tool TSAfinder.^25^ Of note, unlike the approach used in the original study, NovumRNA predictions were solely based on RNA-seq data and did not require the integration of additional mass spectrometry measurements. While useful for confirming expressed/presented peptides, mass spectrometry entails additional costs and logistical challenges, is rarely available next to RNA-seq in clinical settings, and, most importantly, has low sensitivity.

One key challenge in the prediction of ncTSA uniquely from RNA-seq data is the correct translation of aberrant, novel transcripts, which requires the prediction of the exact reading frame (RF) and open reading frame (ORF). For RF prediction, previous studies considered all possible three-frame translations.^19^^,^^25^ However, this approach results in a large number of peptides and likely false positives, which can only be reduced via the integration of MS-confirmed sequences, as done in the Laumont study.^19^ Tools that are solely based on RNA-seq data and consider all possible translation options, such as TSAfinder, inevitably pose a challenge in the prioritization of the most promising targets from this large set of predictions. NovumRNA addresses this issue by applying a primary filtering step already at the nucleotide level, using reference proteins for translation wherever possible and providing the nucleotide sequence of ncTSAs in the output for further frame testing. NovumRNA accurately predicted 96% of all MS-confirmed peptides reported in the Laumont study, but still obtained diverging results in terms of ncTSA due to alternatively called RF and ORF. While our test using BORF,^54^ a recently developed tool for ORF prediction, did not result in a marked improvement, we expect that more accurate tools emerging in the near future can be integrated in NovumRNA to further improve its predictions.

False positive predictions, i.e., putative TSAs that are in reality self-peptides, represent another key challenge in ncTSA prediction performed from tumor RNA-seq data alone. NovumRNA reduces false positives by leveraging a collection of non-cancerous transcript sequences. The pipeline includes an internal control database derived from TEC RNA-seq data, inspired by the Laumont study,^19^ but further allows for the flexible derivation of larger and/or tissue-specific control databases from user-specified RNA-seq data. We demonstrated the value of this approach by using normal colon RNA-seq data from the GTEx project^46^ to ensure the tumor-specificity of ncTSA candidates predicted from CRC organoid data. Even with only 30 GTEx samples, self-peptide candidates were reduced by 43%, demonstrating the effectiveness of even small collections of non-cancerous samples from the same tissue context. The control databases built in this study are available from “https://zenodo.org/records/13642055/files/NovumRNA_resource_bundle.tar.gz?download=1”. Large datasets collections such as GTEx or (healthy samples from) TCGA^36^ offer extensive data which can be used to build more extensive, multi-tissue control databases in NovumRNA. Ultimately, the stringency of the filtering and the resulting trade-off between false positive vs. false negative predictions depend on the specific application and on the available data. Therefore, we made available in NovumRNA a module to easily build a control database from user-supplied healthy RNA-seq data, possibly including both the default TEC samples plus some tissue-specific ones, as we did in the analysis of osteosarcoma data (details in STAR Methods). In the near future, we plan to provide precomputed control databases from different organs and tissues. While we refer to NovumRNA predictions as *tumor-specific* antigens to align with the nomenclature used in Laumont et al.,^19^ we note that the degree of tumor-specificity depends on several factors, including the antigen class (e.g., differential vs. novel), the chosen parameter settings, and the filtering database –each of which should be tailored to the intended application. Most importantly, top ncTSA candidates should be prioritized leveraging the associated metadata provided by NovumRNA (e.g., binding affinity, expression level) and, for clinical applications, validated experimentally.

The identification of new immunotherapy targets from non-canonical tumor aberrations can open new therapeutic avenues for patients with hard-to-treat cancers. Patients with low-TMB, such as individuals with MSS CRC, are currently not benefitting from cancer immunotherapy, despite responses to ICB immunotherapies having been reported in some settings.^14^^,^^15^ NovumRNA analysis of RNA-seq data from ten CRC organoids and 499 tumor samples from the TCGA CRC cohort predicted comparable numbers of novel ncTSAs in MSI and MSS tumors, which would suggest similar potential for the presentation of non-canonical tumor-antigen targets. Top candidates ncTSAs identified by NovumRNA, prioritized based on their level of expression and likelihood of being recognized by T cells, included peptides previously associated with melanoma or with the ERVH-5 gene, in line with previous studies.^65^ Our analysis further identified a set of putative ncTSAs shared across multiple patient-derived organoids, many of which originated from ERV sequences. We further analyzed with NovumRNA an independent cohort from the TARGET initiative (Table S13), encompassing 86 patients with osteosarcoma –a cancer type associated with a low number of canonical neoantigens, poor clinical prognosis, very limited therapeutic opportunities, and no significant clinical progress in the last four decades.^66^^,^^67^^,^^68^^,^^69^ The analysis of this aggressive cancer type analysis also resulted in the identification of several shared ncTSAs (Figure S4). Taken together, these evidences underscore an important feature of ncTSAs: unlike canonical neoantigens, which are rarely shared between patients,^70^ they can potentially enable the development of “off-the-shelf” immunotherapies targeting shared tumor antigens.

Glioblastoma represents another hard-to-treat cancer. This extremely aggressive form of brain tumor has been, so far, intractable with both conventional and immunotherapy approaches due to its low-immunogenic nature and highly immunosuppressive milieu.^71^^,^^72^ Nevertheless, it was reported that patients with high-TMB gliomas, in a context of constitutional DNA mismatch repair deficiency syndrome, can benefit from ICB immunotherapy.^73^^,^^74^ More recently, it was also shown that recurrent glioblastomas carrying low TMB and an enriched inflammatory gene signature can benefit from immunotherapy and survive longer than recurrent glioblastomas with higher TMB.^74^ Motivated by the ncTSA dynamics predicted with NovumRNA on public data from tumor cell lines treated with indisulam, we performed an experiment in glioblastoma primary cell lines perturbed with this splicing-modulating drug. Our investigation included two cell lines derived from recurrent glioblastomas (patient 2 and patient 4) and two from primary glioblastomas. Notably, patient 1 was characterized by an *MGMT*-methylated, hypermethylated, and potentially hypermutated glioblastoma, and previously showed long-term response to dendritic cell (DC) immunotherapy in the DENDR1 trial (NCT04801147).^75^^,^^76^ From this drug-perturbation RNA-seq data, NovumRNA predicted an increase in ncTSAs upon indisulam treatment, with dynamics that were patient-specific. We performed the experimental validation of a selection of indisulam-induced ncTSAs, confirming the immunogenicity of 6 out of 11 peptides (54%) specifically in the indisulam-treated samples. The patients’ characteristics summarized above can support the high percentage of immunogenic neoantigens predicted with NovumRNA in our experiment. Our data, together with previous observations describing the ability of indisulam in inducing a proinflammatory microenvironment and increasing CD8^+^ T cell infiltration,^60^^,^^77^ support the possibility of expanding the repertoire of therapeutically relevant HLA I-restricted neoepitopes, offering new avenues for low-TMB and antigenically heterogeneous tumors such as glioblastomas. More broadly, these results suggest that NovumRNA can be systematically applied to drug-perturbed tumor RNA-seq data to pinpoint therapeutic interventions that might boost the immunogenicity of tumor cells and facilitate T cell recognition.

In conclusion, NovumRNA is a comprehensive and fully automated pipeline for the prediction of different classes of ncTSAs from patients’ tumor RNA-seq data. In this study, we demonstrated the value of NovumRNA for pinpointing new candidate targets for immunotherapy and therapeutic interventions, which could synergize with anticancer (immuno)therapies, facilitating the T cell-mediated recognition of tumor cells. The application of NovumRNA to large cohorts of patients with different cancer types can provide insights into the non-canonical landscape of tumor-specific antigens, ultimately guiding the design of therapies with greater clinical efficacy and broader scope.

## Resource availability

### Lead contact

Further information and requests should be directed to and will be fulfilled by the Lead contact, Francesca Finotello (francesca.finotello@uibk.ac.at).

### Materials availability

This study did not generate new unique materials.

### Data and code availability

The NovumRNA pipeline is available at https://github.com/ComputationalBiomedicineGroup/NovumRNA and from Zenodo (https://doi.org/10.5281/zenodo.13885173). The RNA-seq datasets generated from colorectal cancer organoids and from glioblastoma cell lines are available upon controlled access from the European Genome-Phenome Archive (EGA, https://ega-archive.org/), under accession numbers EGAD50000000962 and EGAD50000000957, respectively.

## Acknowledgments

The computational results presented here have been achieved in part using the LEO HPC infrastructure of the University of Innsbruck. The results shown here are in part based upon data generated by the TCGA Research Network (https://cancergenome.nih.gov). The OS results published here are based upon data generated by the Therapeutically Applicable Research to Generate Effective Treatments (TARGET) initiative, phs000218, managed by the NCI. Information about TARGET can be found at https://www.cancer.gov/ccg/research/genome-sequencing/target/about.

The authors would like to thank Anne Krogsdam and the MultiOmics core facility (Medical University of Innsbruck, Austria) for the support with the tumor organoid sequencing, Benedetta Mazzi (Immuno-hematology and transfusion medicine Unit, San Raffaele Institute) for HLA typing, and Alessandro Gori (SCITEC Institute of Chemical Science and Technology "Giulio Natta," National Research Council- CNR) for the support with the peptide synthesis.

This work was supported by 10.13039/501100000921European Cooperation in Science and Technology (COST) Action “Mye-InfoBank” (CA20117, supported by the EU Framework Program Horizon 2020). MA was supported by the Doctoral scholarship granted from the 10.13039/501100012163University of Innsbruck, Vice-Rectorate for Research. FF was supported by the Austrian Science Fund (FWF) (no. T 974-B30 and FG 2500-B) and by the Oesterreichische Nationalbank (OeNB) (no. 18496). ZT was supported by the 10.13039/501100000781European Research Council (ERC) under the European Union’s Horizon 2020 Research and Innovation Programme (grant agreement no. 786295). SP was partially supported by the 10.13039/501100003196Italian Ministry of Health (RRC). DR and RG are supported by the Austrian Science Fund (FWF) (10.55776/EFP45) under the Emerging Fields Program.

## Author contributions

Conceptualization: FF and MA. Methodology: MA. Software: MA, DR, and RG. Validation: MA, MF, and SP. Formal analysis: MA, DR, and RG. Investigation: MA, DR, MF, RG, GL, RL, SP, ZT, and FF. Resources: FF, ZT, SP, and DR. Data curation: MA, DR, and SP. Writing – original draft: FF, MA, DR, and SP. Writing – review and editing: MA, DR, MF, RG, GL, RL, SP, ZT, and FF. Visualization: MA. Supervision: FF, SP, DR, and ZT. Project administration: FF. Funding acquisition: FF, ZT, SP, and DR.

## Declaration of interests

FF consults for iOnctura.

## STAR★Methods

### Key resources table

**Table:** 

REAGENT or RESOURCE	SOURCE	IDENTIFIER
**Antibodies**		

Mouse monoclonal anti-HLA-ABC (clone W6/32)	Thermo Scientific	Cat# MA5-11723; RRID: AB_10984365
Mouse IgG2a κ isotype control	Thermo Scientific	Cat# 02–6200; RRID: AB_2532955

**Biological samples**		

Primary colorectal tumor tissue	Medical University Hospital of Innsbruck	Table S2
Glioblastoma tumor tissue	Fondazione IRCCS Istituto Neurologico Carlo Besta	Table S10
Blood samples for HLA typing	Fondazione IRCCS Istituto Neurologico Carlo Besta	Table S10

**Chemicals, peptides, and recombinant proteins**		

Indisulam	Sigma Aldrich	SML1225
Human EGF	Peprotech	Cat#AF-100-15
Y27632	AbMole	Cat#M1817
Geltrex	Thermo Scientific	Cat#A1413202
B-27 Supplement	Thermo Scientific	Cat#17504044
GlutaMAX	Thermo Scientific	Cat#3550061
A83-01	Tocris	Cat#2939
SB202190	Sigma	Cat#S7067
Primocin	InvivoGen	Cat#ant-pm-2
N-Acetylcysteine	Sigma	Cat#A9165
IL-2	Miltenyi Biotec	N/A
ERCC RNA Spike-In Mix	Thermo Fisher	Cat#4456740
Puregene Blood Core Kit	Qiagen	Cat# 158389

**Critical commercial assays**		

Nucleospin RNA Mini Kit	Macherey-Nagel	Cat#11912512
TRIzol Reagent	Thermo Fisher	Cat#15596026
RNeasy Mini Kit	Qiagen	Cat#74104
RNase-Free DNase Set	Qiagen	Cat#79254
IFN-γ ELISA	R&D Systems	Cat#DIF50C

**Deposited data**		

NovumRNA source code	This paper	https://github.com/ComputationalBiomedicineGroup/NovumRNA
NovumRNA resource bundle	This paper	https://zenodo.org/records/13642055
CRC organoid RNA-seq	This paper	EGAD50000000962
Glioblastoma RNA-seq	This paper	EGAD50000000957
rMATS cell line RNA-seq	Shen et al.,^52^ 2014	SRA: SRS354082
1000 Genomes RNA-seq for HLA benchmarking	1000 Genomes Project Consortium et al.,^51^ 2015	E-GEUV-1
Laumont et al. validation RNA-seq	Laumont et al.,^19^ 2018	GEO: GSE113972
Healthy colon and brain RNA-seq	GTEx Consortium et al.,^46^ 2017	GTEx portal, Table S4
Treated melanoma cancer cell lines RNA-seq	Lu et al.,^60^ 2021	GEO: GSE162818
Treated bladder cancer cell lines	Ding et al.,^59^ 2016	SRA: SRP063667
TCGA CRC cohort RNA-seq	Cancer Genome Atlas Research Network et al.,^36^ 2013	gdc.cancer.gov, Table S8
Pediatric osteosarcoma RNA-seq	TARGET project via dbGaP/GDC	sbGAP: phs000218
Healthy osteoblast RNA-seq	Moriarity et al.,^78^ 2015	GEO: GSE57925
ERV annotation BED	Paces et al.,^43^ 2002	https://herv.img.cas.cz/
Reannotated GTEx samples	Bastian et al.,^79^ 2021	https://www.bgee.org/support/data-sets

**Experimental models: Cell lines**		

Colorectal cancer organoids	This paper	Table S2
Glioblastoma neurospheres	This paper	Table S10

**Software and algorithms**		

NovumRNA pipeline	This paper	https://github.com/ComputationalBiomedicineGroup/NovumRNA
HISAT2 v.2.1.0	Kim et al.,^37^ 2019	http://ccb.jhu.edu/software/hisat2
StringTie v.2.1.4	Pertea et al.,^38^ 2015	https://ccb.jhu.edu/software/stringtie
Bedtools v.2.26.0	Quinlan and Hall,^80^ 2010	https://bedtools.readthedocs.io/
GFFread v.0.12.3	Pertea and Pertea,^81^ 2020	https://github.com/gpertea/gffread
OptiType v.1.3.3	Szolek et al.,^40^ 2014	https://github.com/FRED-2/OptiType
netMHCpan v.4.1	Jurtz et al.,^82^ 2017	https://services.healthtech.dtu.dk/service.php?NetMHCpan-4.1
netMHCIIpan v.4.0	Reynisson et al.,^42^ 2020	https://services.healthtech.dtu.dk/service.php?NetMHCIIpan-4.0
sam4weblogo.jar	https://doi.org/10.6084/M9.FIGSHARE.1425030.V1	N/A
biostar154220.jar	https://doi.org/10.6084/M9.FIGSHARE.1425030.V1	N/A
sortsamrefname.jar	https://doi.org/10.6084/M9.FIGSHARE.1425030.V1	N/A
SeqKit v.2.0.0	Shen et al.,^83^ 2016	https://bioinf.shenwei.me/seqkit/
YARA v.1.0.2	Dadi et al.,^84^ 2018	https://github.com/seqan/dream_yara
Samtools v.1.12	Li et al.,^29^ 2009	http://www.htslib.org/
pVACtools v.4.0.1	Hundal et al.,^28^ 2020	https://pvactools.readthedocs.io/
IEDB MHCI toolkit v.3.1.2	Vita et al.,^85^ 2018	https://www.iedb.org/
IEDB MHCII toolkit v.3.1.6	Vita et al.,^85^ 2018	https://www.iedb.org/
R v.4.2.3	R Core Team	https://www.r-project.org/
ggplot2 v.3.4.2	Wickham,^86^ 2016	https://ggplot2.tidyverse.org/
Nextflow DSL2 v.23.04.2.5870	Di Tommaso et al.,^35^ 2017	https://www.nextflow.io/
Singularity v. 3.8.7-1.el7	Kurtzer et al.,^45^ 2017	https://sylabs.io/singularity/

### Experimental model and study participant details

#### Colorectal cancer organoids

Histologically verified primary colorectal tumor tissues were obtained from patients undergoing surgical resection at the Medical University Hospital of Innsbruck with the approval of the medical ethical committee of the Medical University of Innsbruck for the establishment of colorectal cancer organoid cultures, protocol AN2016-0194 366/4.9. Written informed consent was obtained from the patients prior to surgical sampling. Samples were obtained from adult female or male patients who were treatment-naïve, with the exception of patient CRC39 who received FOLFOX and cetuximab before surgery. Details regarding the patient’s clinical information are provided in Table S2.

#### Glioblastoma patients and primary cell lines

Glioblastoma samples were provided from patients operated at Fondazione IRCCS Istituto Neurologico Carlo Besta. Written informed consent was obtained from all participants. Clinical data, including age, gender, IDH1 status, and MGMT status are reported in Table S10. Sex (biological) of all participants was recorded and is provided in Table S10. Gender identity, ethnicity, and socioeconomic status were not collected or analyzed, as they were not relevant to the research objectives and were unavailable for most participants.

### Method details

#### The NovumRNA pipeline

NovumRNA is a fully-automated bioinformatic pipeline designed to predict non-canonical tumor-specific antigens (ncTSAs) from raw RNA-seq data. Implemented in the workflow language Nextflow DSL2,^35^ NovumRNA ensures ease of use, maximum reproducibility, portability, and parallelism. The pipeline utilizes Singularity containers,^45^ managed by Nextflow, which eliminates the need for users to install multiple tools and their dependencies manually.

The pipeline takes as input FASTQ files (zipped or unzipped, single-end or paired-end) from human tumor RNA-seq data. The input data must be provided in a comma-separated values (CSV) formatted sample sheet, containing a unique sample identifier (ID), the sequencing read files (“Read1”, “Read2”), and optionally, files containing class-I (“HLA_types”) and/or class-II HLA types (“HLA_types_II”). The sample sheet, as well as other parameters, are specified in the novumRNA config file (“novumRNA.config”). For a detailed description on how to install and use the pipeline, we refer to the NovumRNA documentation on the GitHub repository. Due to licensing restrictions, netMHCpan 4.1 and netMHCIIpan^42^ 4.0 are not shipped with NovumRNA, but they are downloaded from IEDB MHCI toolkit 3.1.2 and MHCII toolkit 3.1.6 and installed on the first run after the user accepts the licenses (“--accept_license”).

Raw sequencing reads are then aligned to the GENCODE reference genome (GRCh38)^39^ using HISAT2^48^ 2.1.0. NovumRNA comes with a pre-built HISAT2 index but can also build indices for the specified reference genome and aligner on the fly. StringTie^38^ 2.1.4 is used to perform reference-guided transcript assembly (“-G gencode.gtf”) based on the aligned reads. A custom python script adds the sample name to the “Transcript ID” in the resulting GTF file and calculates novel features based on the StringTie reported transcript information. Specifically, “isoform_count” is the number of transcript isoforms, counted via the assigned StringTie IDs (e.g., “STRG.1.1”, “STRG.1.2”). “TPM_iso_perc” is the percentage of a transcript’s expression (TPM), relative to the sum of the expression of all isoforms. A high percentage indicates a prevalent abbreviated transcript compared to non abbreviated isoforms, which is crucial to select ncTSAs with higher presentation potential. “Exon_cov_ratio” is the mean transcript read coverage, divided by the read coverage of each individual exon, to prioritize ncTSAs derived from exons similarly supported by reads like the non abbreviated ones.

NovumRNA employs an innovative filtering strategy based on a so-called capture BED file to capture novel and differentially expressed transcript fragments from tumor samples relative to RNA-seq data from healthy tissues. For this, healthy-tissue RNA-seq data are aligned to the same reference, and the transcripts are reconstructed with StringTie (same as tumor); the resulting GTF files are concatenated to a database which is used to build the capture BED file. The capture BED file consists of two parts: putative novel transcript regions and putative differential regions. Putative novel transcript regions are created by inverting coordinates from the healthy database GTF to identify regions with zero coverage in healthy samples. Based on the coordinates, they are labeled intronic (between exons) or intergenic (between genes). Putative differential regions are regions that are sparsely covered in the healthy database. Bedtools^80^ 2.26.0 is used to merge overlapping exon regions from different transcripts in the healthy database GTF; the maximum TPM and maximum coverage from the exons merged together is calculated. By default, regions are considered sparse if the maximum TPM <1 and maximum coverage <4; these cut-offs can be changed by the user. NovumRNA embeds a pre-built default capture BED file based on 32 RNA-seq libraries from 8 samples of human thymic epithelial cells (TECs), originally published by Laumont et al.^19^ and retrieved from the NCBI Gene Expression Omnibus (GEO)^87^ with accession codes GSE127825 and GSE127826. This default capture BED file serves as a ground filtering reference. However, users can build their own file based on available RNA-seq data from healthy tissues. To capture the putative regions, the cancer sample GTF file from StringTie is intersected with the capture BED file using bedtools. The captured regions are filtered using a custom python script, keeping only regions longer than 23 nucleotides, coming from transcripts expressed with at least 1 TPM and covered by ≥ 4 reads for novel ones, and ≥10 and ≥16 for differential ones, respectively; these cut-offs can be changed by the user.

Transcript fragments are then translated into peptides in two steps: translation of the whole transcript based on the StringTie GTF and translation of tumor-specific regions. For full transcript translation, if a known reference protein is available (“gencode.v41”), the transcript is translated in all three reading frames using a custom Python script and the Biopython^88^ package. The frame that has the longest overlap with the reference protein is selected and trimmed at the first stop codon. If no reference protein is available, the most likely open reading frame is chosen either based on the presence of a Kozak motif, or the closest Methionine to the 5′ end. Cancer-specific regions from the overlap BED file are converted to nucleotide FASTA files using GFFread^81^ 0.12.3 and the GENCODE reference genome (GRCh38 version), three-frame translated, and compared to the complete translated transcript. The region showing the highest overlap (minimum of 8 amino acids) is selected and extended to include junction-spanning peptides. A sliding window approach fragments the translated regions into peptides of a specified length, keeping track of the corresponding genomic locations noted in the BED file.

The read coverage of the peptide regions in the original BAM file is checked as an additional quality check, with the BAM file subsetted to retain only high-quality reads. Read coverage is obtained using `sam4weblogo.jar`^89^ (git commit hash: 1ca216453) which outputs patient-specific reads covering the regions, considering only reads fully spanning the peptide region. Regions with high coverage are capped at 100 reads, using `sortsamrefname.jar` (git commit hash: d29b24f2b) and ‘biostar154220.jar’^89^ (git commit hash: d29b24f2b) to expedite analysis, and relevant regions are filtered with a custom python script, keeping peptides covered by at least two high-quality reads (cut-off can be changed by user). Simultaneously, patient-specific SNPs are incorporated into the peptides, previously based only on the reference genome sequence. If the total read count and individual count differ, an SNP is present, and the sequence constituting the majority is selected. The selected nucleotide sequences are once more translated to reflect the SNPs at the peptide level.

NovumRNA also performs filtering on a peptide level. The reference proteome (“gencode.v41”) is split into a FASTA file containing all possible peptides of a specified length using a sliding window approach within a custom python script. Only the novel predicted ncTSAs are filtered against this library of healthy reference peptides. The pipeline comes with a default file containing peptides of 9 and 15 amino acids in length, other lengths are built on the fly, if desired.

In parallel, NovumRNA predicts class I HLA types using OptiType^40^ 1.3.3. Input RNA-seq FASTQ files are mapped using the YARA mapper^84^ 1.0.2 to an HLA RNA reference. The resulting BAM file is used as input for OptiType, which is run in default mode with the “–rna” option. NovumRNA allows users to skip this step by submitting class I HLA types directly as input within the CSV sample sheet. The HLA typing tool HLA-HD,^41^ used for predicting class II HLA types, is not pre-installed with NovumRNA due to licensing constraints and must be installed separately by the user, although it is not mandatory for a successful run. To bypass HLA-HD installation, class II HLA types can be directly added to the input CSV samplesheet.

Inferred HLA types are used to predict the binding %rank of the final ncTSAs surviving all filtering steps using netMHCpan 4.1 or netMHCIIpan^42^ 4.0 part of the IEDB toolkit^85^ utilized by pVACtools^28^ 4.0.1 Only binding peptides are kept: peptides with %rank <2 are labeled as weak binders (WB) and those with %rank <0.5 as strong binders (SB).

Finally, all information and generated metadata are consolidated into the final output TSV tables, including additional annotations —such as intronic, intergenic, and differential— based on a BED file containing known ERV regions in the human genome from HERVd.^43^ NovumRNA also generates a CSV input sample sheet containing result file paths of already run modules, allowing for reruns without repeating resource-intensive steps like alignment.

#### HLA typing benchmark

A gold standard was constructed by leveraging data from two studies that conducted high-precision genotyping of class I (HLA-A, HLA-B, and HLA-C) and class II (HLA-DRB1, HLA-DQB1) HLA types in individuals participating in the 1000 Genomes Project,^49^^,^^50^^,^^51^ like done previously.^24^ RNA-seq data of 247 individuals, consensually called in both studies, was retrieved from ArrayExpress (https://www.ebi.ac.uk/arrayexpress, accession: E-GEUV-1). We selected only individuals having calls for every HLA gene in both studies and, after conversion of all HLA types to four-digit resolution, we defined the final consensus types as the intersection of the HLA types reported by both studies for each individual and HLA gene. Each of the tools inferred HLA allele was compared to the gold standard to identify the number of correct (“match”) and wrong (“mismatch”) calls, as well as percentage with respect to the total possible calls. Comparison statistics and plots for this and all other analyses presented in this article were made with custom R scripts, using R version 4.2.3 and ggplot2 v. 3.4.2.

#### Assessment of exon-inclusion detection and quantification

Raw human RNA-seq data generated in the rMATS study^52^ was obtained from the NCBI Sequence Read Archive (SRA),^90^ under accession code SRS354082. In total, six RNA-seq samples, from two cell lines (PC3E, GS689) with three replicates each, were obtained. Reverse transcription polymerase chain reaction (RT-PCR) validated exon inclusion events, percent spliced in (PSI) values and delta PSI (dPSI) values, i.e., the difference of exon inclusion between two samples/conditions, were obtained from the supplementary material of the original publication. Events genomic coordinates were lifted from the hg19 annotation to the hg38 annotation, using the `hgLiftOver` online tool (https://genome.ucsc.edu/cgi-bin/hgLiftOver).

Events were stored in the BED file format and intersected with the StringTie^38^ assembled GTF derived from NovumRNA analysis of the cell lines, using bedtools intersect.^80^ A custom python script was used to identify transcripts including the exon (“pre_exon”, “exon”, “post_exon”) and transcripts excluding the exon (“pre_exon”, “post_exon”). Excluding transcripts were defined as to specifically show the pre_exon, followed by the post_exon, possible further isoforms are summed up as “others”. For all transcripts including or excluding the exon, the “TPM_iso_perc” was calculated as the percentage of the transcript expression in TPM relative to the sum of the expression in TPM of all transcript isoforms (inclusion + exclusion + others). If multiple transcript isoforms include/exclude the exon, their fractions were summed up. The mean fraction of exon inclusion/exclusion across the three replicates was calculated.

The delta TPM_iso_perc (“dTPM_iso_perc”) was calculated in concordance to the delta PSI (dPSI) values reported in the study (dPSI = PSI_PC3E - PSI_GS689), based on RT-PCR. Pearson correlation was calculated between the average dPSI from the study and NovumRNA’s average “dTPM_iso_perc” per reported exon.

#### Validation of NovumRNA using publicly available data

Raw RNA-seq data generated in the Laumont et al. study^19^ from seven primary human cancer samples (4 B-lineage acute lymphoblastic leukemias and 3 lung cancers) was retrieved from GEO with accession code GSE113972. Mass spectrometry eluted peptides, list of TSAs, healthy control expression, called HLA types and TSAs genomic locations were retrieved from the supplementary materials of the original study. For sample 10H080, peptides from immunopeptidomics and elution were concatenated, and only unique peptide sequences were used as reference.

TSAFinder results on this dataset were obtained from the supplementary material of TSAFinder publication.^25^

NovumRNA analysis was performed using default parameters, considering peptide lengths of 8, 9, 10, and 11 amino acids.

#### Colorectal cancer organoid generation and RNA sequencing

Colorectal cancer organoids were generated and cultured as previously described.^91^ Briefly, freshly isolated tumor cells were seeded in 30 μL droplets of 70% Geltrex (ThermoScientific, Cat#A1413202) on 6 wells-plates (Sarstedt, #83.3920), 4 drops each well, in PDO culture medium: Advanced DMEM/F12 (Thermo Scientific, Cat#12634028) supplemented with 10 mM HEPES solution (Sigma, Cat#H0887), 10 mL/L Penicillin-Streptomycin solution, 2 mM GlutaMAX (Thermo Scientific, Cat#3550061), 20% R-spondin conditioned medium, 10% Noggin conditioned medium, 20 mL/L B-27 supplement (Thermo Scientific, Cat#17504044), 1.25 mM N-Acetylcysteine (Sigma, Cat#A9165), 0.5 nM A83-01 (Tocris, Cat#2939), 10 mM SB202190 (Sigma, Cat#S7067), 50 ng/mL human EGF (Peprotech, Cat#AF-100-15), 100 mg/mL Primocin (Invivogen, Cat#ant-pm-2), and 10 mM Y27632 (AbMole, Cat#M1817). PDO culture medium was replaced every two days. To passage the PDOs, Geltrex droplets were dissociated in TripLE Express (Thermo Scientific, Cat#12604013) and re-plated in 30 μL droplets typically with a 1 to 4 split density. PDOs were harvested, snap-frozen and total RNA was isolated using the Nucleospin Mini Kit (Macherey-Nagel, #11912512) following manufacturer’s instruction and submitted to total transcriptome full-length mRNA sequencing (Genewiz for Azenta Life Sciences, Leipzig, Germany).

#### Analysis of RNA sequencing data from colorectal cancer organoids

Human healthy colon RNA-seq samples were obtained from the GTEx repository^46^ for filtering. GTEx offers in total 686 healthy colon samples. However, the Bgee initiative re-annotated all samples available on GTEx and discarded the ones they deemed unhealthy based on the pathological reports, creating a high-quality subset, restricted to only healthy and non-contaminated samples.^79^ Thereby, the total number of purely healthy colon samples we considered for filtering was 262 (list of sample accession identifiers in Table S3). FASTQ files underwent preprocessing via NovumRNA, employing the “capture_bed” entry to generate corresponding GTF files. Subsequently, input samplesheets were constructed, encompassing randomly selected GTF files. This selection process began with 10 files and iteratively increased in increments of 10, with 10 replicates for each step, ultimately yielding 260 distinct samplesheets. Each of these samplesheets served as input for NovumRNA, utilizing the “capture_bed_short” entry to produce 260 corresponding capture BED files. The resulting capture BED files were employed for filtering in NovumRNA analysis. The input data comprised RNA-seq data obtained from 10 colorectal cancer organoids. We applied NovumRNA with default parameters, considering peptides of 9 amino acid length. Jaccuard similarity was calculated as the ratio between the number of ncTSAs in the replicates over the number of unique ncTSAs across all replicates.

For each of the ten CRC organoids and each GTEx filtering step (ranging from 10 to 260 samples with increments of ten), as well as for the ten replicates performed at each step, we identified putative self-peptides. These were defined as ncTSAs that were not present (i.e., filtered out) when using the full GTEx dataset (262 samples) for filtering. Using the filtering step with 10 GTEx samples as the reference, we calculated the percentage of removed self-peptides at each filtering step, averaging the result across all replicates and organoids, as follows:

Percentage of removed self-peptides = $\frac{{\underline{X}\;}_{10}-{\underline{X}\;}_{i}\;}{{\underline{X}\;}_{10}}$, where:

${\underline{X}\;}_{10}$ = Average number of self-peptides across all organoids and all replicates at filtering with 10 samples.

${\underline{X}\;}_{i}$ = Average number of self-peptides across all organoids and all replicates at filtering with i samples (for i = 10, …,260).

A Wilcoxon rank-sum test (Mann-Whitney U test) was performed to test the number of predicted ncTSAs between MSI and MSS CRC samples.

Top ncTSA candidates across all organoids at maximum filtering (262 GTEx samples) were selected considering only novel ncTSAs from intronic or intergenic source, labelled as strong or weak binder to at least one HLA type, with TPM_iso_perc ≥ 50%, TPM >3, BAM_reads >5, and Exon_cov_ratio ≥ 0.8. From these, only one ncTSA per transcript with the strongest binding affinity was chosen.

To assess the shared status of the ncTSAs, we considered only peptides derived from transcripts composed of more than one exon, as determined by StringTie transcript assembly.

Seven public antigen databases^55^^,^^56^^,^^57^^,^^92^^,^^93^^,^^94^ (https://caped.icp.ucl.ac.be/) were exported to text files. ncTSAs from 10 CRC organoids, filtered with 262 GTEx samples, were queried in the exported files. ncTSAs needed to be contained in the peptide sequence reported by the database; no mismatch was tolerated.

Predicted ERV derived ncTSA nucleotide sequences, taken from output column “REF_NT”, were used as query and searched in a related nucleotide sequence for gene ERVH-5, taken from AL138920.11 (50173.52469), as reported here https://www.ncbi.nlm.nih.gov/gene/100862699. The search was performed using the SeqKit tool^83^ v. 2.0.0 with the `locate` command.

The colon capture_BED file is available as part of the NovumRNA resource bundle: “https://zenodo.org/records/15537803/files/NovumRNA_resource_bundle.tar.gz?download=1”.

#### Analysis of tumor RNA sequencing data from The Cancer Genome Atlas and TARGET

Human CRC RNA-seq data from TCGA was obtained from the database of Genotypes and Phenotypes (dbGaP)^95^ via the Genomic Data Commons (GDC).^96^ A total of 499 CRC samples with available MSI or MSS status (Table S8), extracted from The Cancer Immunome Atlas (TCIA https://tcia.at),^70^ were included; “MSI-L” and “MSI-H” samples were considered as “MSI”. BAM files were coverted into FASTQ format and were analyzed via NovumRNA to predict ncTSAs, using the capture BED file based on 262 healthy colon GTEx samples for filtering.

RNA-seq data from 86 human pediatric osteosarcoma (OS) patients available in the TARGET project were obtained from dbGaP via GDC (phs000218, Table S13). BAM files were converted into FASTQ format and processed with NovumRNA. An OS-specific capture BED file was generated using NovumRNA’s built-in pipeline with default settings, including the “default” TEC data as well as RNA-seq data from three normal human osteoblast samples^78^ (GEO accessions: GSE57925, GSE127825 and GSE127826).

The combined osteoblast and TEC capture_BED file is available as part of the NovumRNA resource bundle: “https://zenodo.org/records/15537803/files/NovumRNA_resource_bundle.tar.gz?download=1”.

To assess the shared status of the ncTSAs, we considered only peptides derived from transcripts composed of more than one exon, as determined by StringTie transcript assembly.

Comparative analyses of the average number of predicted ncTSAs between MSI and MSS samples were conducted using the Wilcoxon rank-sum test, with Bonferroni correction applied for multiple comparisons. Statistical analyses and visualizations were performed using R, with visualizations generated through ggplot2.

#### Analysis of public RNA sequencing data from treated cancer cell lines

Raw RNA-seq data from human cancer cell lines that had been subjected to treatments influencing gene transcription were retrieved from two independent studies from GEO with accession GSE162818, and SRA with accession SRP063667. Subsequently, NovumRNA was employed to analyze each individual sample using default parameters, considering peptides 9 amino acids long. A one-sided Wilcoxon rank-sum test (Mann-Whitney U test) was performed, using alternative = “greater”, to test if the number of predicted ncTSAs of treated (indisulam, MS023) is greater than the control (DMSO).

#### Glioblastoma patients’ characteristics

MGMT promoter methylation status was assessed by methylation-specific polymerase chain reaction using specific primers.^97^ IDH1 gene mutation detection was performed using primer pairs specific for exon 4 and determined by Sanger sequencing (Applied Biosystems 3500 and 3500 Dx Series Genetic Analyzer). HLA typing was performed using polymerase chain reaction (PCR) using sequence-specific oligonucleotides (PCR-SSO) at Immuno-hematology and transfusion medicine Unit (San Raffaele Institute) on DNA extracted from blood samples (Puregene Blood Core Kit, Qiagen).

#### Generation of RNA sequencing data from glioblastoma primary cell lines treated with indisulam

Four glioblastoma primary cell lines (BT592/pt1, BT1007/pt2, BT1009/pt3; BT1012/pt4) were derived from glioblastoma Cavitron Ultrasonic Aspirator (CUSA) material as previously described^98^^,^^99^ and cultured as neurospheres (GB-NS) in DMEM/F12+Glutamax medium containing B27 supplement (Thermofisher, Waltham, MA, USA) and the mitogenic factors epidermal growth factor (EGF) and fibroblast growth factor b (bFGF) (Peprotech, Rocky Hill, NJ, USA). GB-NS were confirmed mycoplasma-free by PCR Test. Cells were seeded in triplicate in 6-well plates, treated with 5 μM indisulam (Sigma Aldrich, Merk), or vehicle control, and collected after 6 and 16 h for RNA extraction.

Total RNA was extracted using TRIzol Reagent (Life Technologies, Thermo Fisher, cat n. 15596026), RNeasy Mini Kit, and RNase-Free DNase Set (Qiagen, cat n. 74104 and 79254). Sample QC, RNA library preparations, and sequencing reactions were conducted at GENEWIZ (Germany GmbH). The concentration of RNA was quantified using a Qubit Fluorometer (Life Technologies, Carlsbad, CA) and RNA integrity was assayed using a TapeStation. Sequencing libraries were prepared with RNA with PolyA selection and ERCC spike-in with standard ERCC kit (ERCC RNA Spike-In Mix, Thermo Fisher 4456740). Final libraries (Illumina, RNA with PolyA selection) were sequenced on a NovaSeq platform, producing 2 × 150 base paired-end reads.

#### Analysis of RNA sequencing data from glioblastoma cell lines treated with indisulam and selection of top candidates for validation

NovumRNA was employed to analyze each individual sample using a capture_BED file built based on 120 healthy brain GTEx samples deemed purely healthy by the bgee suite^79^ (list of sample accession identifiers in Table S3), predicting peptides of 9 amino acids length.

Predicted peptides submitted for validation from patients 1, 2, and 4, were selected based on desirable metadata features: Predicted only after indisulam treatment, so not found in the DMSO control, only strong binders (%rank <0.5), TPM_iso_perc = 100, Exon_cov_ratio ≥ 0.8 and TPM ≥ 1, BAM_reads ≥ 5. From the remaining pool of peptides fulfilling these features, only one peptide per transcript was selected based on the lowest %rank score. From these, four peptides per patient with the lowest %rank were chosen. For patient 1, a peptide was selected which occurred with 6 h and 16 h indisulam treatment, leading to three individual peptides validated.

The brain capture_BED file is available as part of the NovumRNA resource bundle: “https://zenodo.org/records/15537803/files/NovumRNA_resource_bundle.tar.gz?download=1”.

#### Experimental validation via co-culture experiments

A total of 2x10^5^ autologous dendritic cells (DCs) were plated in 24 well-plates, pre-loaded with 10 μg/mL of each selected peptide (synthetized by Primm) for 2 h at 37°C and then co-cultured with 2x10^6^ PBMCs (ratio 1:10 = DC:PBMCs) for three days in a final volume of 2 mL RPMI 1640 supplemented with 10% human serum, 100 U/ml penicillin, 100 U/ml streptomycin, 100 μg/mL glutamine, 0.1 mM non-essential amino acids, 1 mM sodium pyruvate, 50 μM β-mercaptoethanol the presence of 30 U/ml of IL-2 (Miltenyi Biotec, Germany).

Pre-stimulated PBMCs were co-cultured in the presence of GB-NS properly treated with indisulam or DMSO as control (E:T = 3:1) in GB-NS medium without serum and in the presence of B27 supplement. Co-culture supernatants were collected after 24 h to measure IFN-γ using specific ELISA (R&D system, Minneapolis, MN). The memory status of T cells was assessed by using CD45RA and CD62L that distinguish stem memory/naive (CD45RA^+^ CD62L^+^); effector memory (CD45RA^−^ CD62L^−^); central memory (CD45RA− CD62L^+^). For MHC class-I blocking assay, anti-HLA-ABC monoclonal antibody (W6/32, Thermo Scientific) or IgG2a kappa control isotype (Thermo Scientific) were added to indisulam-treated-GB-NS at 20 μg/mL for 30 min before adding prestimulated patient-derived PBMCs for co-culture.

A one-sided Wilcoxon rank-sum test (Mann-Whitney U test) was performed, using alternative = “greater”, to test for increased secretion of IFN-γ for pre-stimulated PBMCs and GB-NS cells treated with indisulam or DMSO, compared to the control for evaluated peptides (Table S11). The same approach was used to test for increased IFN-γ secretion in HLA-unblocked versus HLA-blocked conditions in GB-NS cells treated with indisulam.

### Quantification and statistical analysis

All statistical analyses were performed using custom R scripts (R v4.2.3) with visualizations generated using ggplot2 v3.4.2. HLA typing performance was assessed by comparing inferred alleles to a gold standard, calculating match and mismatch rates. Exon inclusion was evaluated by correlating delta TPM_iso_perc values from NovumRNA with delta PSI values derived from RT-PCR using Pearson correlation. Group comparisons of predicted ncTSA counts between MSI and MSS CRC samples, as well as treatment versus control (indisulam, MS023 vs. DMSO), were conducted using Wilcoxon rank-sum tests, with Bonferroni correction applied for multiple comparisons. Additional one-sided Wilcoxon rank-sum tests were used to assess increased IFN-γ secretion in co-culture assays involving peptide-pulsed PBMCs and glioblastoma neurospheres, including comparisons between HLA-blocked and unblocked conditions.

## Supplemental information

Document S1. Figures S1–S4 and Tables S1, S2, S4, S10, and S12

Table S3. List of GTEx samples used to build the colon and brain control databases

Table S5. Top non-canonical tumor-specific antigens (ncTSA) candidates predicted from colorectal cancer organoid RNA sequencing data

Table S6. Non-canonical tumor-specific antigens (ncTSA) from colorectal cancer organoids matching public antigen databases

Table S7. Non-canonical tumor-specific antigens (ncTSA) shared across different colorectal cancer organoids

Table S8. List of patients with colorectal cancer from The Cancer Genome Atlas (TCGA) program considered in the analysis

Table S9. Non-canonical tumor-specific antigens (ncTSA) shared across different patients with colorectal cancer from The Cancer Genome Atlas (TCGA)

Table S11. Top non-canonical tumor-specific antigens (ncTSA) candidates predicted from glioblastoma cell lines treated with indisulam

Table S13. List of patients with osteosarcoma cancer from the TARGET initiative considered in the analysis
