# Supplementary material for: NovumRNA: Accurate prediction of non-canonical tumor antigens from RNA sequencing data
Source: iScience. 2025 Sep 3;28(10):113448. doi: 10.1016/j.isci.2025.113448 (PMC12478123; doi:10.1016/j.isci.2025.113448)
Supplement: Document S1. Figures S1–S4 and Tables S1, S2, S4, S10, and S12 [file mmc1.pdf]

iScience, Volume *28*

Supplemental information

NovumRNA: Accurate prediction of non-canonical tumor antigens from RNA sequencing data

Markus Ausserhofer, Dietmar Rieder, Manuel Facciolla, Raphael Gronauer, Giorgia Lamberti, Rebecca Lisandrelli, Serena Pellegatta, Zlatko Trajanoski, and Francesca Finotello

Supplementary tables

Table S1 | Summary of tools for non-canonical tumor-specific antigen (ncTSA) prediction

Overview of key features of computational pipelines for ncTSA identification, including antigen sources, supported Major Histocompatibility Complex (MHC) classes, and input data types. “Standalone tool” indicates whether the method runs independently; “Perform data preprocessing” refers to built-in support for upstream tasks like alignment or variant calling; “HLA typing” shows whether HLA alleles are inferred or must be provided. “Experimentally validate” indicates evidence from experimental assays; “Immunogenicity score” refers to in silico prediction of T-cell recognition. HLA: Human leukocyte antigen.

Tool Name	Tool type	ncTSA source	class-I	class-II	Standalone tool	Perform data preprocessing	HLA typing	Last Update	Experimen-tally validated	Immunogenicity score	Input data type	Reference
NeoFuse	Shell and Python (containerized)	Gene-fusions	Yes	No	Yes	Yes	Yes	2022	No	No	RNA-seq FASTQ files	Fotakis et al., 2020
pVACfuse	Python	Gene-fusions	Yes	Yes	No	No (e.g. AGFusion needs to be run first)	No (HLA types as input)	2025	No	Yes (DeepImmuno)	Ouptut from fusion prediction tools	Hundal et al., 2020
INTEGRATE-NEO	C++ and Python	Gene-fusions	Yes	No	Yes	Yes	Yes	2018	No	No	RNA-seq FASTQ files	Zhang et al., 2017
NeoSplice	Python	Alternative-splicing	Yes	No	No	No (Requires aligned reads and HLA types)	No (HLA types as input)	2021	Yes (Mass spectrometry confirmation)	No	Tumor and normal control RNA-seq BAM files	Chai et al., 2022
pVACsplice	Python	Alternative-splicing	Yes	Yes	No	No (Requires RegTools output)	No (HLA types as input)	2025	No	Yes (DeepImmuno)	Annotated VCF file and a RegTools output file (tsv)	Richters et al., 2022
Splice2Neo	R package	Alternative-splicing	No (NeoFox is used)	No (NeoFox is used)	No	No (Requires splicing variants, variant calling, HLA types)	No (HLA types as input)	2025	Yes (qRT-PCR confirmed exon skipping junctions)	No	Output from diverse splicing tools (WES and RNA-seq based)	Lang et al., 2024

SNAF	Python package	Alternative-splicing	Yes	No	No	No (Requires aligned reads and HLA types)	No (HLA types as input)	2024	Yes (Mass spectrometry confirmation)	Yes (DeepImmuno)	RNA-seq BAM files	Li et al., 2024
TSAFinder	Python	Filter approach, multiple	Yes (requires netMHCpan)	No	No	Yes	Yes (requires seq2HLA)	2021	No	No	Tumor and control RNA-seq FASTQ files	Sharpnack et al., 2022
NeoDisc	Shell and Python and R (containerized)	Proteogenomic approach, multiple	Yes	Yes	No (substantial extra installations required)	Yes	Yes (requires HLA-HD)	2025	Yes (T-cell immunogenicity essay)	No	Tumor and control WES/WGS and tumor RNA-seq FASTQ files	Huber et al., 2024
nextNEOpi	Nextflow DLS1 (containerized)	Uses NeoFuse and pVACsplice	Yes	Yes	Yes	Yes	Yes (requires HLA-HD)	2025	No	Yes (CSiN immunogenicity score, DeepImmuno)	Tumor and control WES/WGS and tumor RNA-seq FASTQ files	Rieder et al., 2022
**NovumRNA**	Nextflow DLS2 (containerized)	Filter approach, multiple	Yes	Yes	Yes	Yes	Yes (requires HLA-HD)	2025	Yes (T-cell immunogenicity essay)	No	Tumor and control RNA-seq FASTQ files	This study

Table S2 | Colorectal cancer organoid metadata information

Sample identifier, gender, and age of the patient, as well as subtype (as microsatellite instable, MSI, or microsatellite stable, MSS) and anatomical location of the tumor.

ID	Gender	Age	Subtype	Tumor location
CRC01	male	75	MSI	ascending colon
CRC13	female	90	MSI	sigmoid colon
CRC22	male	87	MSI	ascending colon
CRC34	male	52	unknown	sigmoid colon
CRC35	female	74	MSS	sigmoid colon
CRC39	male	44	MSS	sigmoid colon
CRC41	female	37	MSS	sigmoid colon
CRC42	male	60	MSS	sigmoid colon
CRC43	female	68	MSS	cecum
CRC44	male	58	MSS	sigmoid colon

Table S4 | False positive peptides from the Laumont study

This table reports the number of false-positive (FP) peptides from the Laumont study, i.e., not confirmed as tumor-associated antigens (TSAs), for each sample. Additionally, it presents the number of non-canonical TSA (ncTSAs) from NovumRNA matching these FP peptides, further broken down into "differential" or "novel" ncTSAs.

Sample	Laumont FP peptides	NovumRNA total matches (FP)	NovumRNA matches differential (FP)	NovumRNA matches novel (FP)
07H103	800	1	1	0
10H080	3,106	5	5	0
10H118	167	0	0	0
12H018	369	1	1	0
lc2	5,281	4	4	0
lc4	4,615	6	6	0
lc6	2,886	6	6	0

Table S10 | Glioblastoma patients' metadata information.

Patient identifier, sample identifier, diagnosis, age at surgery, gender, IDH1 and MGMT status. GB-NS: glioblastoma neurospheres; IDH: Isocitrate dehydrogenase; MGMT: O-6-methylguanine-DNA methyltransferase.

Pt ID	GB-NS ID	Diagnosis	Age at surgery	Gender	IDH1	MGMT methylation
Patient 1*	BT592	glioblastoma	45	M	WT	MET (0.74)
Patient 2	BT1007	recurrent glioblastoma	59	M	WT	UNMET (0.00)
Patient 3	BT1009	glioblastoma	77	M	WT	UNMET (0.00)
Patient 4	BT1012	recurrent glioblastoma	76	F	WT	MET (2.48)

*Patient 1 is Pt23 in DENDR1 clinical trial (NCT04801147).

Table S12 | Memory of T cells after peptide stimulation

Patient/sample identifier, peptide and treatment information, percentages of CD8+ T-cell subsets; stem memory/naïve: CD45RA+ CD62L+; effector memory: CD45RA− CD62L−; central memory: CD45RA− CD62L+

Patient	Peptide	Treatment	%CD8+ T stem memory/naive	%CD8+ T central memory	%CD8+ T effector memory
Patient 1/ BT592	unstimulated	vehicle	17.51	23.01	38.61
		Indisulam	19.40	30.94	36.18
	IIAKCIQLK	vehicle	21.91	46.59	29.91
		Indisulam	18.02	43.81	25.59
	VPVPTTPCV	vehicle	27.80	44.94	21.95
		Indisulam	23.00	45.75	31.24
	**IPVYACGPV**	**vehicle**	**25.61**	**48.11**	**31.46**
		**Indisulam**	**10.20**	**38.72**	**71.20**
Patient 2 / BT1007	unstimulated	vehicle	25.6	32.4	33.8
		Indisulam	24.9	33.9	35.3
	KTILGNMMK	vehicle	15.4	34.7	33.2
		Indisulam	17.5	36.1	38.4
	LQFCNFKSV	vehicle	17.5	31.8	37.3
		Indisulam	14.7	32.8	36.7
	**WQQGSFFLL**	**vehicle**	**19.9**	**38.2**	**33.9**
		**Indisulam**	**9.8**	**36**	**50.1**
	**TAVSPWVEL**	**vehicle**	**28.7**	**28.6**	**41.7**
		**Indisulam**	**10.2**	**39.7**	**51.2**
Patient 4 / BT1012	unstimulated	vehicle	23.8	29.5	42.9
		Indisulam	22.6	33.3	42.6
	**MGDGARLCL**	**vehicle**	**26.9**	**32.1**	**22.1**
		**Indisulam**	**13.7**	**22.3**	**55.3**
	RMAHMSRTF	vehicle	20.8	44.8	31.9
		Indisulam	23.3	42.7	31.2
	**FVVPPQGAL**	**vehicle**	**20.3**	**29.4**	**30.53**
		**Indisulam**	**12.4**	**26.4**	**42.1**
	**LARNSPLFL**	**vehicle**	**21.1**	**41.4**	**29.9**
		**Indisulam**	**13.4**	**43.2**	**40.7**

Supplementary figures

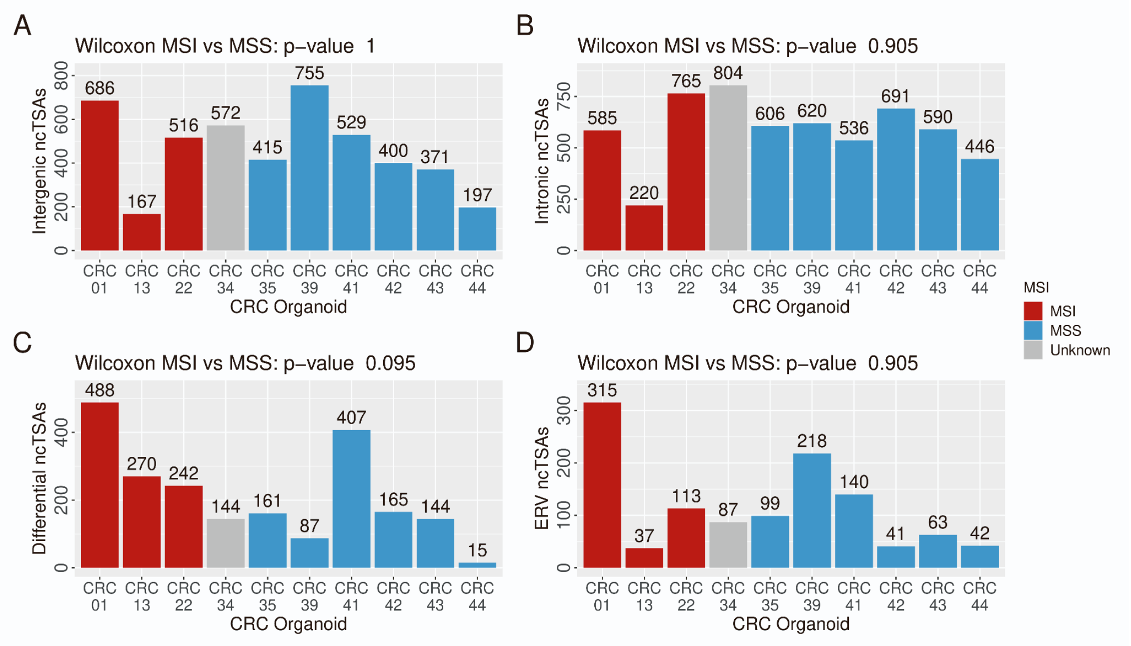

Figure S1 | NovumRNA predicted ncTSAs in MSI versus MSS CRC organoids.
Number of non-canonical tumor-associated antigens (ncTSAs) of different origin predicted by NovumRNA per colorectal cancer (CRC) organoid, colored by the organoid subtype. MSI: microsatellite instable; MSS: microsatellite stable; ERV: endogenous retrovirus.

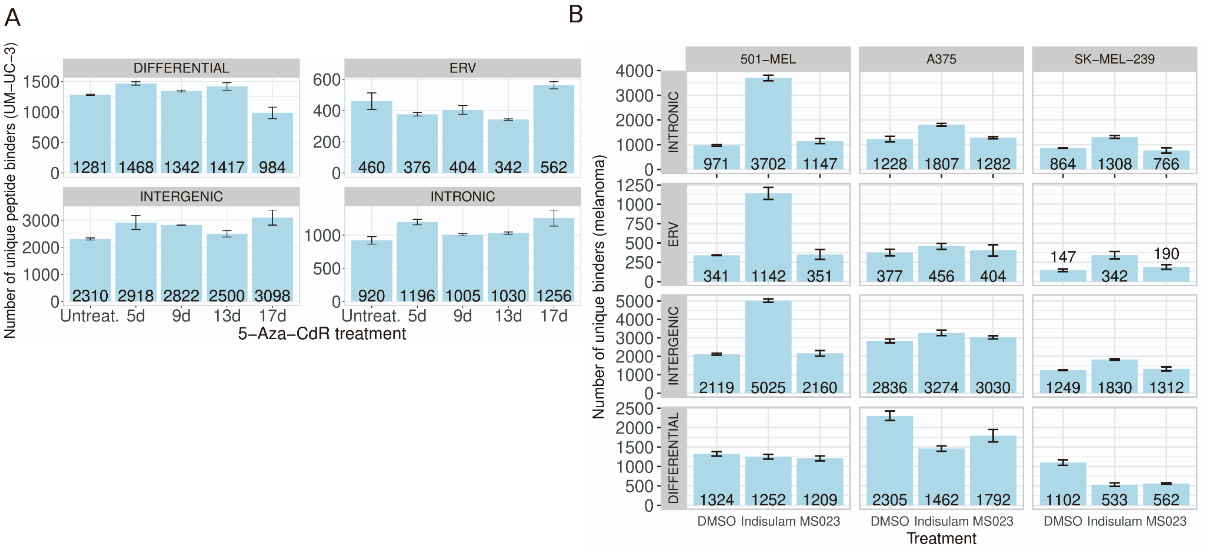

Figure S2 | Predicted non-canonical tumor-associated antigens (ncTSAs) in splicing-perturbed human cell lines
A) The bar plot shows the mean number of unique ncTSAs predicted by NovumRNA for human cell line UM-UC-3, treated with splicing-perturbation drug 5-Aza-CdR. Samples were taken on days 5, 9, 13, and 17. Each treatment was performed with two biological replicates, error bars indicate the standard deviation. **B)** Bar plot shows the mean number of unique ncTSAs predicted by NovumRNA for three human cell lines, 501 MEL, A375, SK MEL 239, treated with splicing-perturbation drugs Indisulam and MS023, while DMSO was used as a control. Each treatment was performed with three biological replicates, error bars indicate the standard deviation. ERV: endogenous retrovirus.

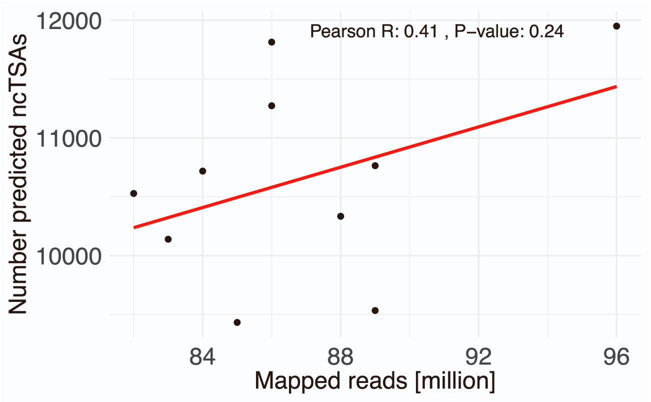

Figure S3 | Pearson correlation of aligned reads and number of non-canonical tumor-associated antigens (ncTSAs) predicted with NovumRNA from the Ding dataset

The x-axis shows the number of mapped reads (in millions) in the UM-UC-3 cell line data from Ding et al. The x-axis shows the number of non-canonical tumor-associated antigens (ncTSAs) predicted for each sample by NovumRNA.

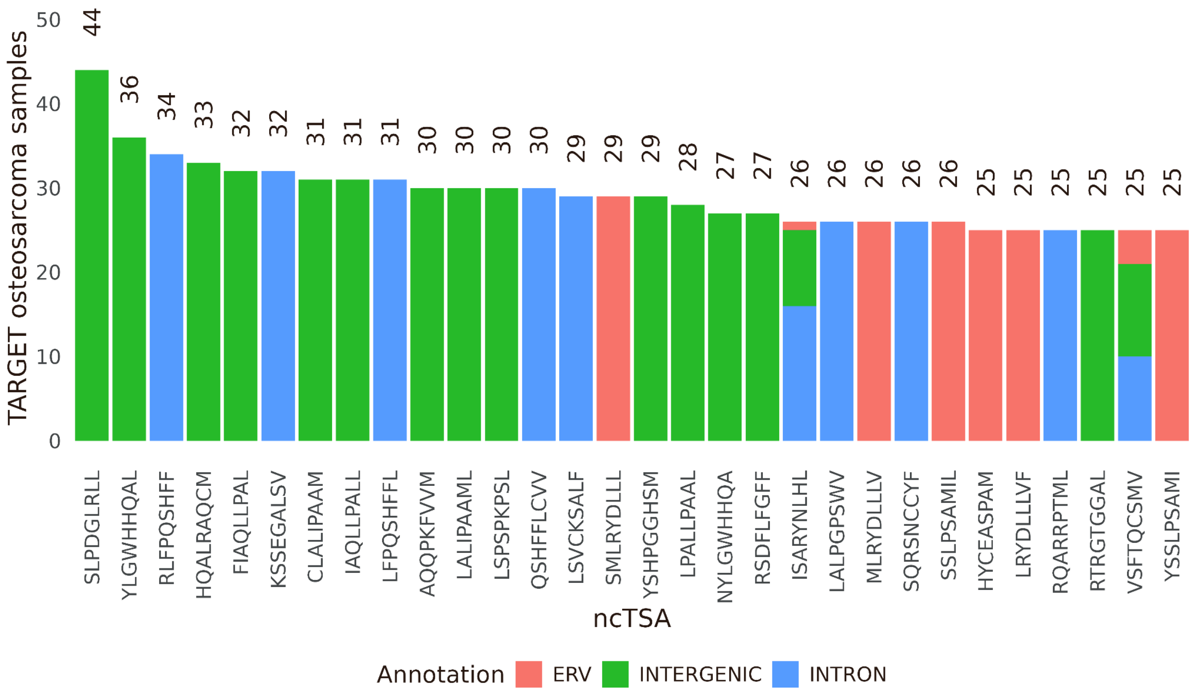

Figure S4 | Top-shared non-canonical tumor-associated antigens (ncTSAs) in the TARGET osteosarcoma patient cohort.

Top 30 most shared ncTSAs predicted by NovumRNA from the TARGET osteosarcoma patient cohort (n=86). Bars show how many samples share each peptide, colored by their origin annotation. Considered were only ncTSAs derived from multi-exon transcripts. TARGET: Therapeutically Applicable Research to Generate Effective Treatments; ERV: endogenous retrovirus.
